# Investigation, scaffold hopping of novel donepezil-based compounds as anti-Alzhiemer’s agents: synthesis, in-silico and pharmacological evaluations

**DOI:** 10.1038/s41598-024-51713-4

**Published:** 2024-01-19

**Authors:** Mohan Gupta, Swati Pant, Preeti Rana, Avinash Kumar, Chakrawarti Prasun, Maya S. Nair, Sarvesh Paliwal, Sumitra Nain

**Affiliations:** 1 Department of Pharmacy, Banasthali Vidyapith Newai, Banasthali, Rajasthan India; 2 Department of Medicinal Chemistry, National Institute for Pharmaceutical Education and Research (NIPER) Balangar, Hyderabad, India; 3 Department of Medical Affairs, Curie Sciences Pvt. Ltd., Samastipur, Bihar 848125 India; 4 Department of Biosciences and Bioengineering, Indian Institute of Technology Roorkee, Roorkee, Uttarakhand 247667 India

**Keywords:** Learning and memory, Drug discovery, Neuroscience

## Abstract

Alzheimer’s disease (AD) is a multifaceted neurodegenerative condition. The pathogenesis of AD is highly intricate and the disease is apparent in the aged population ~ 50–70 years old. Even after > 100 years of research, the root origin of AD and its pathogenesis is unclear, complex and multifaceted. Herein, we have designed and synthesized 9 novel molecules with three different heterocyclic scaffolds namely pyrrolidone-2-one, quinoline & indoline-2-one to imitate and explore the novel chemical space around donepezil. The synthesized molecules were evaluated for their potential as anti-Alzheimer’s agents through *in-vitro* and *in-vivo* studies in appropriate animal models. To further understand their interaction with acetylcholinesterase enzyme (AChE), extra-precision docking, and molecular dynamics simulation studies were carried out. As the number of compounds was limited to thoroughly explore the structure–activity relationship, atom-based 3D-quantitative structure–activity relationships (QSAR) studies were carried out to get more insights. All the designed compounds were found to inhibit AChE with IC_50_ in the micromolar range. From pyrrolidone-2-one series, 6-chloro-N-(1-(1-(3,4-dimethoxybenzyl)-2-oxopyrrolidin-3-yl)piperidin-4-yl)pyridine-3-sulfonamide (**9**), 2-(1-benzylpiperidin-4-yl)-6,7-dimethoxy-4-(4-methoxyphenyl)quinoline (**18**) from quinoline series and N-(1-benzylpiperidin-4-yl)-2-(2-oxoindolin-3-yl)acetamide (**23**) from indolin-2-one series inhibited AChE with an IC_50_ value of 0.01 µM. Based on other biochemical studies like lipid peroxidation, reduced glutathione, superoxide dismutase, catalase, nitrite, and behavioural studies (Morris water maze), compound **9** was found to be a potent AChE inhibitor which can be further explored as a lead molecule to design more potent and effective anti-Alzheimer’s agents.

## Introduction

Alzheimer’s disease (AD) is a multifaceted neurodegenerative condition. The pathogenesis of AD is highly intricate and the disease is apparent in the aged population ~ 50–70 years old^1^. At present globally, AD has been diagnosed in around 50 million people and it is expected to double every 5 years. As per the Lancet Commission (2020), there will be nearly 150 million AD patients by 2050, with the impacts of AD costing the global economy over US$1 trillion^2^. Even after > 100 years of research, the root source of AD is unknown, except few symptomatic treatments mainly cholinesterase inhibitors^1^. The cholinergic, amyloid, and tau hypothesis each contribute to the pathophysiology of AD. Drugs like tacrine (discontinued in 2013), memantine, rivastigmine, galantamine, and donepezil have been used for symptomatic treatment of AD. Out of these donepezil which is an acetylcholinesterase (AChE) inhibitor, has become the preferred choice for the management of AD^3^.

Several research groups have reported donepezil-based compounds as AChE inhibitors with potent anti-AD activity. Modifications have largely been carried out either at the dimethoxy-substituted indanone ring the methylene linker or the N-benzylpiperidine ring system of donepezil. Several heterocyclic scaffolds like pyrrolidines, quinolines, pyrazoles etc. have been attached to donepezil to explore the chemical space around donepezil^4^. Previously our group also reported some novel donepezil-based pyrrolidine-2-one/indolin-2-one derivatives as AChE inhibitors with potent anti-AD activity^3^. Wang et al. reported donepezil-based 8-hydroxyquinoline derivatives as potent AChE inhibitors^5^. Cai et al. have also joined donepezil with 4-N-phenylaminoquinoline to discover novel AChE inhibitors^6^. Based on our work and prior literature reports, we have continued our search for novel donepezil-based derivatives as anti-AD agents.

Herein, we have designed (see Supplementary Fig. S1 online) and synthesized 9 novel molecules with three different heterocyclic scaffolds namely pyrrolidone-2-one, quinoline & indoline-2-one to emulate and explore the novel chemical space around donepezil. The synthesized molecules were evaluated for their potential as anti-Alzheimer’s agents through in-vitro and in-vivo studies in appropriate animal models. To further understand their interaction with acetylcholinesterase enzyme (AChE), extra-precision docking, and molecular dynamics simulation studies were also carried out. As the number of compounds was limited to properly explore the structure–activity relationships, atom-based 3D-quantitative structure–activity relationships (QSAR) studies were carried out to get some insights.

## Material and methods

### Materials

All key raw materials and solvents were of laboratory grade procured from Merch, Combi-blocks, and Enamine and used as such for the synthesis without purification. The reactions were monitored with the help of TLC (Thin layer chromatography) using silica gel pre-coated plates (60F254, Merck, 0.25 mm thickness) and visualized in ultraviolet (UV) light or by using staining solutions like potassium permanganate &amp; Ninhydrin solutions. The purifications were performed by gravity column chromatography over silica gel (230–400 mesh size) packed in glass columns and indicated solvent systems. The percentage yields reported are based on pure products and are not optimized. 1H- and 13C-NMR spectra were recorded on Bruker Avance II 400 MHz spectrometer in DMSO-d6 solvent at 400 MHz and 100 MHz, respectively. All chemical shifts (δ) are expressed in parts per million (ppm) relative to the standard TMS and the peak patterns are indicated as (s) singlet, (d) doublet, (t) triplet, (m) multiplet, and (br) broad signal. Mass spectra are recorded using a Thermo Fisher mass spectrometer with an electron ionization technique^4^.

### Chemistry

**Figure 1:**
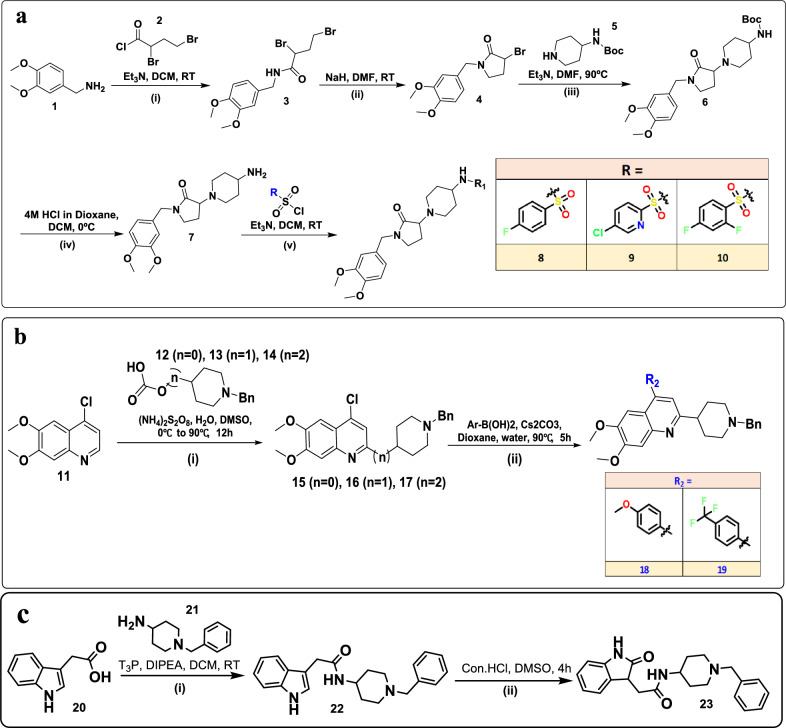
(**a**) Scheme 1: (i) Et_3_N, DCM, 0 °C to RT, 2 h (ii) NaH, DMF, 0? to RT, 30 min. (iii) Et_3_N, DMF, 90?, 5 h (iv) 4 M HCl in Dioxane, DCM, RT, 1 h. (v) Et_3_N, Ar-Sulfonyl chloride, 0?, 1 h. (**b**) Scheme 2: (i) (NH_4_)_2_S_2_O_8_, H_2_O, DMSO, 90?, 12 h (ii) Ar-B(OH)_2_, Cs_2_CO_3_, Dioxane, water, 90?, 5 h. (**c**) Scheme 3: (i) T3P, DIPEA, DCM, 0?, 5 h (ii) Con HCl, DMSO, 4 h.

Built on the prior literature reports, an assorted collection of 9 novel molecules was designed and prepared synthetically to imitate and explore the novel chemical space around the lead molecule Donepezil. The scheme-1 sulfonamide triade of molecules (Fig. 1a) is synthesized from our lead Intermediate-4 by coupling with N-boc 4-amino piperidine, the intermediate so isolated was further BOC-deprotected and coupled with three readily available sulfonyl chlorides to generate 8, 9, 10. The scheme 2 quinoline series (Fig. 1b) is outlined by scaffold hopping of indanone core of donepezil with 6,7-dimethoxyquinoline analogues. The KSM (4-chloro-6,7-dimethoxyquinoline) was reacted with Oxalic acid esters of piperidine alcohols (varied chain length) under modified Minisi reaction conditions to afford **15, 16 & 17**. One of these intermediates i.e., **15** was further subjected to Suzuki reaction with 4-Methoxy phenyl boronic acid and 4-CF3 phenyl boronic acid to afford compounds like 18 and 19. A unique indole molecule **23** is synthesized as per Scheme 3 (Fig. 1c) to bring in some novel interactions with support of Molecular design ideas and synthesized smoothly by coupling bench top available intermediate-21 using peptide coupling reagent T3P to get Int-22 which was further oxidized to achieve compound **23**.

#### Synthesis of 2,4-dibromo-N-(3,4-dimethoxybenzyl)butanamide (3)

To a stirred solution of (3,4-dimethoxyphenyl)methanamine **1** (1 g, 5.98 mmol, 1 eq) in DCM (20 mL), triethyl amine (4.2 mL, 29. 9 mmol, 5 eq) at 0 °C under nitrogen atmosphere added 2,4 dibromo butyryl chloride **2** (1.19 mL, 8.97 mmol, 1.5 eq) and continued stirring at room temperature for 30 min. The reaction mixture was diluted with Sat.NH4Cl (50 mL) and extracted with DCM (2 × 50 mL). The combined organic layer was dried over Na2SO4 and evaporated under reduced pressure to get crude residue. The crude was purified over a silica gel column eluted with 50% Ethyl acetate in Hexane to get desired product 3 (pale yellow colour liquid; 1.7 g; Yield: 71%). 1H-NMR: (400 MHz, DMSO-d6): δ 8.89 (s, 1H, NH), 6.90–6.77 (m, 3H, ArH's), 4.60 (t, J = 8 Hz, 1H, CH-CO), 4.31–4.17 (m, 2H, CH2), 3.90 (s, 6H, OCH3), 3.65–3.50 (m, 3H, CH2-Br) and 2.42–2.32 (m, 2H, CH2-CH); MS (ESI): m/z calculated for C13H17Br2NO3 [M+H] + 392.96, found 395.93.

#### Synthesis of 3-bromo-1-(3,4-dimethoxybenzyl)pyrrolidin-2-one (4)

To a stirred solution of 2,4-dibromo-N-(3,4-dimethoxybenzyl)butanamide **3** (1.7 g, 4.33 mmol) in DMF (30 mL) added NaH (260 mg, 6.45 mmol) and stirred at same temperature for 1 h. After completion of the reaction by TLC, the reaction mixture was diluted with brine solution (30 mL) and extracted with ethyl acetate (50 mL × 2). The combined organic layer was dried over Na_2_SO_4_ and evaporated under reduced pressure to give a crude residue, which was purified by a gravity silica column using ~ 60% ethyl acetate in hexane to get the desired product as pale-yellow liquid **4** (Pale yellow liquid; 1 g; Yield: 73.97). ^1^H-NMR: (400 MHz, DMSO-d6): δ 6.93 (d, J = 8 Hz, 1H, ArH), 6.80–6.76 (m, 2H, ArH’s), 4.73 (d, J = 7.2 Hz, 1H, CH-Br), 4.48 (d, J = 12 Hz, 1H, Ar-CH2), 4.25 (d, J = 16 Hz, 1H, Ar-CH2), 3.74 (s, 6H, OCH_3_), 3.26–3.21 (m, 2H, N-CH2-CH2), 2.73–2.59 (m, 1H, CH2-CH-Br) and 2.20–2.16 (m, 1H, CH2-CH-Br); MS (ESI): m/z calculated for C_13_H_16_BrNO_3_ [M+H]^+^ 313.03, found 314.08.

#### Synthesis of tert-butyl (1-(1-(3,4-dimethoxybenzyl)-2-oxopyrrolidin-3-yl)piperidin-4-yl)carbamate (6)

To a stirred solution of 3-bromo-1-(3,4-dimethoxybenzyl)pyrrolidin-2-one **4** (2.5 g, 7.9 mmol, 1 eq), tert-butyl piperidin-4-ylcarbamate **5** (2.39 g, 11.9 mmol) in DMF (30 mL) added DIPEA (6.95 mL, 49 mmol) and raised to temperature to 90 °C for 2 h. After completion of the reaction, the reaction mixture was diluted with sat. NaHCO_3_ sol and extracted with ethyl acetate (2 × 100 mL). The combined organic layer was dried over Na_2_SO_4_ and evaporated under reduced pressure to get crude residue. The crude was purified over a silica column eluted with ~ 70% Ethyl acetate in hexane to get the desired product **6** as pale-yellow liquid (3 g, Yield: 86%). ^1^H-NMR: (400 MHz, DMSO-d_6_): δ 7.95 (s, 1H), 6.91–6.89 (d, J = 8 Hz, 1H), 6.75–6.71 (m, 2H), 4.38 (d, J = 12 Hz, 1H), 4.19 (d, J = 16 Hz, 1H), 3.72 (s, 1H), 3.70 (s, 1H), 3.43 (m, 2H), 3.17–3.06 (m, 3H), 2.88 (s, 3H), 2.72 (s, 2H), 2.17–1.99 (m, 2H), 1.83–1.65 (m, 3H) and 1.37 (s, 9H). MS (ESI): m/z calculated for C_23_H_35_N_3_O_5_ [M+H]^+^ 433.55, found 434.5.

#### Synthesis of 3-(4-aminopiperidin-1-yl)-1-(3,4-dimethoxybenzyl)pyrrolidin-2-one hydrochloride salt (7)

To a stirred solution of tert-butyl (1-(1-(3,4-dimethoxybenzyl)-2-oxopyrrolidin-3-yl)piperidin-4-yl)carbamate **6** (3 g, 6.9 mmol, 1 eq) in DCM (20 mL) added 4 M HCl in dioxane (17.5 mL, 70 mmol) at 0 °C and stirred at RT for 30 min. After completion of the reaction, the reaction mixture was evaporated under reduced pressure and triturated with diethyl ether (2 × 10 mL), dried under reduced pressure to get desired product **7** as HCl salt (1.8 g, Yield: 70%). ^1^H-NMR: (400 MHz, DMSO-d_6_): δ 8.46 (bs, 3H), 7.95 (s,1H), 6.92–6.69 (m, 3H), 4.45–4.26 (m, 3H), 4.029 (d, J = 8 Hz, 1H), 3.32–3.12 (m, 4H), 3.12 (bs, 1H), 2.88 (s, 3H), 2.72 (s, 3H), 2.38–2.29 (m, 2H) and 2.14–2.00 (m, 4H). MS (ESI): m/z calculated for C_18_H_28_ClN_3_O_3_ [M+H]^+^ 369.18, found 370.5

### General procedure A

#### Synthesis of N-(1-(1-(3,4-dimethoxybenzyl)-2-oxopyrrolidin-3-yl)piperidin-4-yl)-4-fluorobenzenesulfonamide (8)

To a stirred solution of 3-(4-aminopiperidin-1-yl)-1-(3,4-dimethoxybenzyl)pyrrolidin-2-one hydrochloride **7** (300 mg, 8.1 mmol, 1 eq) in DCM (10 mL) added DIPEA (700 µL, 4.6 mmol) followed by added 4-fluoro benzenesulfonylchloride (185 mg, 9.7 mmol, 1.2 eq) and continued stirring at RT for 16 h. After completion of the reaction, the reaction mixture was diluted with water and extracted with DCM (2 × 10 mL). The combined organic layer was dried over Na_2_SO_4_ and evaporated under reduced pressure to get the desired product **8** as an off-white solid (120 mg, Yield: 30%). ^1^H-NMR: (400 MHz, DMSO-*d*_*6*_): δ 7.88–7.85 (m, 2H), 7.77 (d, J = 8 Hz, 1H), 7.45–7.40 (t, J = 8 Hz, 2H), 6.89–6.87 (d, J = 8 Hz, 1H), 6.73–6.69 (m, 2H), 4.36 (d, J = 12 Hz, 1H), 4.16 (d, J = 12 Hz, 1H), 3.71 (s, 3H), 3.86 (s, 3H), 3.39–3.34 (m, 1H), 3.07–3.03 (m, 2H), 2.89–2.79 (m, 2H), 2.12 (t, J = 12 Hz, 1H), 1.96–1.95 (m, 1H), 1.48 (m, 2H) and1.33 (m, 2H). MS (ESI): m/z calculated for C_24_H_30_FN_3_O_5_S [M+H]^+^ 491.19, found 420.2

#### 6-chloro-N-(1-(1-(3,4-dimethoxybenzyl)-2-oxopyrrolidin-3-yl)piperidin-4-yl)pyridine-3-sulfonamide (9)

^1^H-NMR: (400 MHz, DMSO-d_6_): δ 8.79 (s, 1H), 8.23 (d, J = 8 Hz, 1H), 8.21 (d, J = 2.4 Hz, 1H), 7.78 (d, J = 8 Hz, 1H), 6.89–6.87 (m, 1H), 6.73–6.9 (m, 2H), 4.33–4.13 (m, 2H), 3.72 (s, 3H), 3.68 (s, 3H), 3.40 (t, J = 8 Hz, 1H), 3.09 -3.02 (m, 5H), 2.14–1.96 (m, 3H), 1.80–1.77 (m, 1H), 1.55–1.45 (m, 2H) and 1.35–1.33 (m, 2H). MS (ESI): m/z calculated for C_23_H_29_ClN_4_O_5_S [M+H]^+^ 508.15, found 509.25.

#### N-(1-(1-(3,4-dimethoxybenzyl)-2-oxopyrrolidin-3-yl)piperidin-4-yl)-2,4-difluorobenzenesulfonamide (10)

^1^H-NMR: (400 MHz, DMSO-d_6_): δ: 8.11 (d, J = 7.6 Hz, 1H), 7.09 (q, J = 8.4 Hz, 1H), 7.57 (t, J = 8 Hz, 1H), 7.29 (t, J = 8 Hz, 1H), 6.89 (d, J = 6 Hz, 1H), 6.73–6.69 (m, 2H), 4.36–4.12 (m, 2H), 3.71 (s, 3H), 3.68 (s, 3H), 3.40–3.37 (m, 1H), 3.07–2.96 (m, 3H), 2.83 (d, J = 12 Hz, 1H), 2.12–2.07 (m, 1H), 1.97–1.96 (m, 1H), 1.82–1.75 (m, 1H) and 1.51–1.38 (m, 4H); MS (ESI): m/z calculated for C_24_H_29_F_2_N_3_O_5_S [M+H]^+^ 509.18, found 510.25.

### General procedure-B

#### Synthesis of 2-(1-benzylpiperidin-4-yl)-4-chloro-6,7-dimethoxyquinoline (15)

To a stirred solution of 4-chloro-6,7-dimethoxyquinoline **11** (2.5 g, 11.2 mmol, 1.0 eq), 2-((1-benzylpiperidin-4-yl)oxy)-2- oxoacetic acid **12** (5.89 g, 22.3 mmol, 2 eq) {prepared from corresponding alcohols} (NH_4_)_2_S_2_O_8_ (5.1 g, 22.3 mmol, 2.0 eq), 20.0 mL of DMSO, and 5.0 mL of H_2_O. The reaction mixture was sealed with a PTFE cap and then stirred rapidly at 90 °C for 24 h. After completion of the reaction, the reaction mixture was diluted with 20 mL of 1 M Aqu. NaHCO_3_ solution and extracted with DCM (3 × 20 mL). The combined organic extracts were washed with brine (40 mL), dried over Na_2_SO_4_, and concentrated under reduced pressure to get crude residue, which was purified over a silica column eluted with 50% Ethyl acetate in hexane to get desired product as off-white solid **15** (2 g, Yield: 45%). 1H-NMR: (400 MHz, DMSO-*d*_*6*_): δ 7.50 (s, 1H), 7.37 (s, 1H), 7.33–7.31 (m, 5H), 7.26–7.23 (m, 1H), 3.94 (s, 3H), 3.93 (s, 3H), 3.50 (s, 2H), 2.94 (dd, J = 12 Hz, 2H), 2.80–2.74 (m, 1H), 2.10–2.02 (m, 2H) and 1.87–1.82 (m, 4H). MS (ESI): m/z calculated for C_23_H_25_ClN_2_O_2_ [M+H]^+^ 396.16, found 397.07 (M+1)^+^

#### 1 2-((1-benzylpiperidin-4-yl)methyl)-4-chloro-6,7-dimethoxyquinoline (16)

^1^H-NMR: (400 MHz, DMSO-*d*_*6*_): δ 7.46–7.25 (m, 8H), 3.93 (s, 6H), 3.62 (bs, 1H), 3.50 (bs, 1H), 3.13 (s, 1H), 2.77 (d, J = 8 Hz, 2H), 1.86 (bs, 3H), 1.56 (bs, 2H) and 1.25–1.14 (m, 3H). MS (ESI): m/z calculated for C_24_H_27_ClN_2_O_2_ [M+H]^+^ 410.18, found 411.09 (M+1)^+^

#### 2-(2-(1-benzylpiperidin-4-yl)ethyl)-4-chloro-6,7-dimethoxyquinoline (17)

^1^H-NMR: (400 MHz, DMSO-*d*_*6*_): δ 7.48 (s, 1H), 7.38 (s, 1H), 7.32–7.28 (m, 6H), 3.98 (s, 6H), 3.41 (s, 2H), 2.86–2.77 (m, 5H), 1.87 (m, 2H), 1.67 (m, 4H) and 1.23–1.17 (m, 2H). MS (ESI): m/z calculated for C_25_H_29_ClN_2_O_2_ [M+H]^+^ 424.19, found 425.02 (M+1)^+^

### General procedure-H

#### 2-(1-benzylpiperidin-4-yl)-6,7-dimethoxy-4-(4-methoxyphenyl)quinoline (18)

To a stirred solution of 2-(1-benzylpiperidin-4-yl)-4-chloro-6,7-dimethoxyquinoline **15** (300 mg, 0.75 mmol, 1 eq), (4-methoxyphenyl)boronic acid (175 mg, 0.11 mmol, 1.5 eq) in Dioxane (10 mL), water (5 mL) added Cs_2_CO_3_ (500 mg, 0.15 mmol, 2 eq) and purged the reaction mixture with nitrogen for 2 min to remove dissolved oxygen content, later added Pd(dppf)Cl_2_ (44 mg, 0.06 mmol, 0.08 eq) and sealed with PTFE cap. The reaction mixture was stirred at 100 °C for 5 h. After completion of the reaction, the reaction mixture was diluted with Sat.NH_4_Cl solution and extracted with ethyl acetate (2 × 50 mL). The combined organic layer was dried over Na_2_SO_4_ and evaporated under reduced pressure to get crude residue, which was purified over a silica column eluted with ~ 10% MeOH in DCM to get desired product **18** as brown colour solid (80 mg, Yield: 23%). ^1^H-NMR: (400 MHz, DMSO-*d*_*6*_): δ 7.54 (d, J = 8 Hz, 2H), 7.37–7.33 (m, 5H), 7.28–7.26 (m, 1H), 7.15–7.10 (m, 4H), 3.93 (s, 3H), 3.87 (s, 3H), 3.73 (s, 3H), 3.51 (bs, 2H), 2.95–2.93 (m, 2H), 2.81–2.79 (m, 1H), 2.08 (bs, 2H) and 1.89 (bs, 4H). MS (ESI): m/z calculated for C_30_H_32_N_2_O_3_ [M+H]^+^ 468.24, found 469.09 (M+1)^+^

#### 2-(1-benzylpiperidin-4-yl)-6,7-dimethoxy-4-(4-(trifluoromethyl)phenyl)quinoline (19)

^1^H-NMR: (400 MHz, DMSO-*d*_*6*_): δ 7.93 (d, J = 8 Hz, 2H), 7.84 (d, J = 8 Hz, 2H), 7.61–7.50 (m, 1H), 7.41 (s, 1H), 7.39–7.41 (m, 4H), 7.25 (s, 2H), 7.04 (s, 1H), 3.95 (s, 3H), 3.73 (s, 3H), 3.51 (bs, 2H), 2.96–2.84 (m, 3H), 2.09 (bs, 2H) and 2.00–1.91 (m, 4H). MS (ESI): m/z calculated for C_30_H_29_F_3_N_2_O_2_ [M+H]^+^ 506.22, found 507.07 (M+1).

### Synthesis of N-(1-benzylpiperidin-4-yl)-2-(1H-indol-3-yl)acetamide (22)

To a stirred solution of 2-(1H-indol-3-yl)acetic acid 2-(1H-indol-3-yl)acetic acid **20** (1 g, 5.7 mmol, 1 eq) and 1-benzylpiperidin-4-amine **21** (2.17 g, 11.42 mmol, 2 eq) in DCM (20 mL) added T_3_P (6.54 mL (50% sol in EA), 14.24 mmol, 2.5 eq) followed by added DIPEA (6.54 mL, 40 mmol, 7 eq) and stirred the reaction at RT for 5 h. After completion of the reaction, the reaction mixture was diluted with sat. NH_4_Cl sol (25 mL) and extracted with ethyl acetate (2 × 25 mL). The combined organic layer was dried over Na2SO4 and evaporated under reduced pressure to get crude residue. The crude was further purified over a silica column eluted with 80% EA in hexanes to get the desired product as off-white solid **22** (800 mg, Yield: 40%). ^1^H-NMR: (400 MHz, DMSO-d6): δ 10.82 (s, 1H), 7.89 (d, J = 8 Hz, 1H), 7.54 (d, J = 8 Hz, 1H), 7.33–7.23 (m, 6H), 7.15 (s, 1H), 7.06 (t, J = 8 Hz, 1H), 6.97 (t, J = 8 HZ, 1H), 3.51 (bs, 1H), 3.46 (s, 2H), 3.33 (s, 2H), 2.73–2.70 (m, 2H), 1.99–1.97 (m, 2H), 1.7–1.75 (m, 2H), 1.45–1.40 (m, 2H); MS (ESI): m/z calculated for C_22_H_25_N_3_O [M+H]^+^ 347.24, found 348.24 (M+1)^+^.

### Synthesis of N-(1-benzylpiperidin-4-yl)-2-(2-oxoindolin-3-yl)acetamide (23)

To a stirred solution of N-(1-benzylpiperidin-4-yl)-2-(1H-indol-3-yl)acetamide **22** (800 mg, 2.30 mmol, 1 eq) in DMSO (5 mL) added Con.HCl (3 mL) and stirred the reaction mixture at RT for 4 h. After completion of the reaction (Monitored by TLC), the reaction mixture was evaporated under reduced pressure and diluted with water (30 mL). The precipitated solids were filtered, washed with chilled diethyl ether (2 × 5 mL) and dried under reduced pressure to get the desired product as a pale-yellow solid **23** (350 mg, Yield: 45%). ^1^H-NMR: (400 MHz, DMSO-d6): δ 7.91 (bs, 1H), 7.31 (m, 6H), 7.13–7.08 (m, 2H), 6.90 (m, 1H), 6.8 (d, J = 8 Hz, 1H), 3.65 (bs, 1H), 3.54 (bs, 1H), 3.42 (s, 2H), 2.71–2.68 (m, 3H), 2,37–2.33 (m, 1H), 1.99 (q, J = 12H, 2H), 1.78–1.61 (m, 2H), 1.39–1.28 (m, 2H); MS (ESI): m/z calculated for C_22_H_25_N_3_O_2_ [M+H]^+^ 363.46, found 364.17 (M+1)^+^.

### Biological evaluation

#### Experimental

##### Animals

Swiss albino male mice weighing 25–30 g were purchased from LLRUVAS in Hisar, Haryana, India. All experiments were performed after IAEC's clearance of the protocol (BV/IAEC/4278/2021) and all experiments were perfermod according to OECD and ARRIVE guidelines The mice were housed in groups of six in the standard lab setting (25 ± 2 °C; 60 ± 2% RH) with free access to food and drink (12 h light/dark cycle). The housing, care, and handling of the animals were done per CPCSEA requirements. The animals were exposed to the laboratory setting for seven days before the experiment.

##### Drugs

Donepezil (acetylcholinesterase inhibitor) and scopolamine (cognitive defects inducer) were purchased from Merck (Merck KGaA, Darmstadt, Germany). All other chemicals procured for the experiment were of analytical grade.

##### Administration of dose

The animals were properly acclimated before being randomly placed into 12 groups (I-XII), with six animals in each group. Scopolamine (i.p.) was given for 5 days, at least 2 h before evaluation, as well as in conjunction with donepezil (peroral) and synthetic compounds (peroral). Group I (vehicle only), Group II (scopolamine, i.p., 1.5 mg/kg), Group III (scopolamine mixed with donepezil, p.o., 2.0 mg/kg), and Group IV-XII (scopolamine combined with synthetic compounds 8, 9, 10, 15, 16, 17, 18, 19 and 23 p.o., 2.0 mg/kg). Following the delivery of the dose to the mouse, the rodent's behaviour was assessed using the traditional Morris water maze (MWM) technique.

#### Behavioral studies

##### Morris water maze

Five days of continuous animal evaluations were conducted using the MWM model. It consists of a circular water tank (120 cm × 60 cm; interior painted black) filled to a height of 55 cm with water that is (25 ± 2 °C)^7^. The tank was placed in a dim laboratory, and using wires anchored at the pool's edge at an angle to one another, it was divided into four equal pieces (P1–P4). A stage in the targeted area (P4) of the pool with an upper face (6 × 6 cm^2^) immersed in the water (1.0 cm below the surface). The location of the stage stayed the same throughout the evaluation. The animals were subjected to water pools throughout the evaluation by turning their heads towards the wall from any of the four segments, namely P1-P4, and being given 90 s to recognise the stage placed in a pool. If the animal couldn't get to the stage in 90 s, they were gently guided onto the stage under supervision. On the sixth day, one of the parameters used to determine if cognitive impairments have recovered was the time it took the mice to travel from the initial portion to locate the hidden stage in the intended part (escape latency).

#### Biochemical analysis

After five days of behavioral assessment, the mice's brains were removed and homogenized (blended) in chilled phosphate buffer (pH 7.4) at a concentration of 10% w/v (weight per volume). The homogenates were then centrifuged (spun at high speed) for 20 min at 4 °C to separate the supernatant (liquid) from the pellet (solid material). The supernatant was then collected to measure biological substances.

##### Acetylcholinesterase (AChE)

Employing Ellman et al., methodology, AChE levels were quantified in tissue homogenate supernatant (THS) to evaluate cholinergic abnormalities. The chromophore's molar extinction coefficient (ε = 1.36 × 10^4^ M^−1^ cm^−1^) was calculated and results were shown as moles of AChE per minute per milligram of protein^8,9^.

##### Lipid peroxidation (LPO)

The amount of malondialdehyde (MDA), a marker of lipid peroxidation (LPO), was measured using a method described by Wills^10^. Tris–HCl buffer and THS were mixed and incubated at 37 °C for 2 h with 10% trichloroacetic acid. After incubation, the mixture was centrifuged again (at 1000 rpm for 10 min). Supernatant and 0.67% thiobarbituric acid were added, and the sample tubes were boiled for 10 min. After cooling, 1.0 mL of double-distilled water was added, and the absorbance was measured at 532 nm. The amount of MDA was calculated using an extinction coefficient of 1.56 × 105 M^−1^ cm^−1^ and reported as moles of MDA per milligram of protein.

##### Reduced glutathione (GSH)

The method described by Jollow et al. was used to measure glutathione (GSH) levels. First, 1.0 mL of 4% sulfosalicylic acid was added to 1.0 mL of tissue homogenate to precipitate the GSH. The mixture was then incubated at 4 °C for 1 h and centrifuged at 1200 rpm for 10 min at 4 °C. The supernatant was collected and diluted to a final volume of 3.0 mL with phosphate buffer and Ellman's reagent. The absorbance of the yellow-colored solution was immediately measured at 412 nm, and the results were expressed as nanomoles of GSH per milligram of protein^11^.

##### Superoxide dismutase (SOD)

The samples contained EDTA, sodium carbonate, and NBT, and the assay was performed using Kono's method. Each sample tube contained 2.0 mL of this mixture, as well as THS and hydroxylamine. The absorbance of the mixture was measured at 560 nm every 30 or 60 s for 2 min to track changes in absorbance^12^.

##### Catalase

Employing the methodology of Luck et al., catalase activity was quantified. The sample contained H_2_O_2_ (1.0 mL, 0.019 M), phosphate buffer (1.95 mL, 0.05 M, pH 7.0), and THS (0.05 mL). The optical density was computed at a wavelength of 240 nm. The findings were shown as μM H_2_O_2_ decomposition/min/mg protein^13^.

##### Nitrite

The amount of nitric oxide in THS was calculated using Greiss' reagent, a mixture of chemicals that reacts with nitric oxide to produce a colored product. The absorbance of the colored product was computed at 540 nm and the concentration of nitrite was determined using a standard curve of known nitrite concentrations^14^.

#### Statistical analysis

The obtained results are presented as mean ± SD. Data were evaluated by one-way analysis of variance (ANOVA). All statistical analysis was executed with the help of Graph Pad Prism 8.3.0 software. Statistical significance was set at p < 0.05.

### In-silico studies

All computational studies except MD simulation studies were carried out using Schrodinger 2022-1 Maestro version 13.1.141 on an HP desktop system with an 11th-generation Intel Core processor 2.5 GHz. MD simulation studies were carried out using GROMACS 2018.3 on the LINUX system with NVIDIA GPU support.

#### Molecular docking study

The ligands and protein were prepared before docking using the Glide module of Maestro. To prepare the ligands, all synthesized compounds were drawn in 2D using the Maestro sketcher tool. Then, the LigPrep module was used to convert them to their lowest-energy 3D structures^15^. The OPLS3e force field was used to prepare both the ligands and the protein^16^.

The protein was prepared using the protein preparation wizard tool. The X-ray crystal structure of human AChE in complex with donepezil was downloaded from the Protein Data Bank (https://www.rcsb.org/structure/4EY7) and minimized using the OPLS3e force field using a three-step workflow^17,18^. The receptor grid was generated using the receptor grid generation tool in the Glide module, with donepezil selected as the reference ligand. We selected the donepezil binding site as the active site for our synthesized molecules because we were trying to design AChE inhibitors. The potential for nonpolar parts of the receptor was softened using Van der Waals radii scaling, with a scaling factor of 1.0 and a partial charge cutoff of 0.25^19^.

After docking, all of the docked poses were subjected to free binding energy calculations using the Prime module of the Maestro suite. The MM-GBSA (Molecular Mechanics, the Generalized Born model and Solvent Accessibility) calculations were performed on the docked complexes using the Prime module in Maestro. The ligands were ranked based on their MM-GBSA Dg bind scores. The VSGB 2.0 (Variable dielectric surface generalized Born model) was used as the solvent model for this study.

#### Drug-likeness analysis

In silico absorption, distribution, metabolism, excretion, and toxicity (ADMET) prediction has led to reduced attrition of compounds in later stages of drug discovery^20^. Hence, we have predicted the ADMET properties of our synthesized compounds using the QikProp module in the Maestro panel. QikProp predicts several relevant physical descriptors and employs them for ADMET calculations. Several parameters like ADME-compliance score (indicated by #stars), polar surface area, ionization potential, partition coefficient, absorption, Lipinski rule violations etc. were computed^21^.

#### MD simulation study

MD simulation study was carried out to understand the interaction of the most potent compounds with AChE using GROMACS (version 2018.3)^22^. AMBER ff99SBILDN was employed as the force field while TIP3P was used as the solvent model. The ligand topology was generated using the Antechamber tool of AMBERTOOLS. The solvated complex underwent energy minimization using the conjugate gradient and steepest descent (SD) algorithms. Following minimization, a two-phase equilibration process was performed, and the equilibrated complex was then subjected to a 100 ns MD simulation at 300 K and 1 bar with a time step of 2 fs^18^.

#### Atom-based 3D-QSAR study

As the number of compounds was limited to properly explore the structure–activity relationships, atom-based 3D-quantitative structure–activity relationships (QSAR) studies were carried out to get some insights. The phase module of the Maestro suite was employed for this study^23^. A total of 60 compounds were taken to develop the QSAR model. The ligands showed AChE inhibition activity over three log order ranges and were all donepezil-based derivatives. Apart from the 9 ligands from the present study, compounds were taken from three different sources as shown in Supplementary Table S1^3,24,25^. Although the ligands were taken from different sources to have structural diversity, the assay protocol for the AChE inhibition study was the same for all. Eighty percent of the ligands were used as a training set while twenty percent was used as the test set. For some ligands, the IC_50_ value was in nM while for some it was in µM. Hence, to have uniformity, all values were converted into µM and then further conversion of IC_50_ to pIC_50_ was done by taking the negative logarithm of IC_50_. The partial least square (PLS) factor was fixed at 5 for the model. Leave-one-out (LOO) method was employed for internal validation while the test set molecules served as an external validation set. Several statistical parameters like R^2^, cross-validated R2 (R^2^ CV), Q^2^, SD, p-value, R^2^ scramble, stability, F-value, RMSE, and Pearson-r were computed to test the robustness, relaibility and predicitive ability of the model. To further understand the atom-type contribution towards activity, H-bond donor, hydrophobic/non-polar, negative ionic, positive ionic, and electron-withdrawing contribution of atoms were computed for the activve and inactive molecules. This excersise was undetaken to gain some insights regarding the chemical features which might contribute towards the activty of the molecules reported in this study^26^.

## Results and discussion

### Design strategy

AChE is a hydrolase that catalyses the breakdown of the neurotransmitter ACh and so plays a highly important role in cholinergic transmission^27^. According to previously published crystallographic studies, the active site of AChE comprises a steep narrow ~ 20 Å gorge with two binding sites; one being the catalytic activity site (CAS) at the bottom of the gorge and the other being the peripheral anion site (PAS) at the entrance of the gorge. Many studies reveal that dual i.e. CAS & PAS binding AChE inhibitors delay aggregation of Aβ which generates toxic plaques along with effective inhibition of AChE. Many classical AChE inhibitors have three vital parts which include a core-ring structure that interacts with amino acid residues of PAS, a basic motif (mostly piperidine or piperazine variants) to interact with the aromatic residues of CAS, and a linker to connect these two components, which lies in a narrow part of the active site of AChE^27^. In the pursuit of effective anti-Alzheimer agents, several strategies have been explored using novel compounds based on donepezil. Recent studies have reported that carbazole-based derivatives^28^, benzothiazole–piperazine hybrids^29^, and donepezil-based multi-functional agents^30^ have exhibited potent anti-AD activity through AChE inhibition, Aβ disaggregation, antioxidant properties, and neuroprotective effects. Consequently, in line with our previous findings and a couple of contemporary literature, we have bio-sterically designed a set of 9 compounds with three scaffolds, mimicking the features of donepezil’s core-indanone motif using scaffold hopping software^31–33^.

### Chemistry

After a couple of trials and fine-tuning of stoichiometric calculations, the reactions to synthesize three different scaffolds were optimised through which nine novel chemical entities were synthesized. The Pyrrolidin-2-one (scheme-1) series is based on our earlier efforts which eased to synthesize intermediate-7, which further supported us in preparing final compounds **8, 9** and **10** using corresponding commercially purchased sulfonyl chlorides. The 2nd series (Scheme-2) was built on C-2 alkylation of dimethoxy-quinoline using Ammonium persulfate mediated Minisci-reaction^39^ to synthesize targets **15, 16, 17, 18 and 19**. The last compound **23** was prepared by trivial amidation followed by oxidation of indole to indolin-2-one. All these final compounds are characterized by appropriate analytical data (MS and ^1^H-NMR).

### Biological evaluation

#### Behavioral study

The newly synthesized compounds 8, 9, 10, 15, 16, 17, 18, 19 and 23 (2 mg/kg, oral) and standard compound donepezil (2 mg/kg, oral) on scopolamine-induced memory impairment and learning disability in mice were studied for neuroprotective effects using Morris water maze model^34–36^. The results were compared with only scopolamine-treated animals. The values are shown as mean ± SEM, and results are expressed as escape latency and TSTQ (time spent in the target quadrant) in the Morris water maze model (Table 1).

**Table 1:** TSTQ data of compounds 10a-c, 18a-d, 23a-c and 25a-e.

Compound	Escape latency (s)					TSTQ (s)
	1st Day	2nd Day	3rd Day	4th Day	5th Day	
Control	79.14 ± 1.9	50 ± 1.54	33 ± 2.12	19.23 ± 2.20	9.15 ± 0.99	36.40 ± 0.22
Scopolamine	78.65 ± 0.82	80.64 ± 3.91	82.82 ± 3.79	84.15 ± 3.31	86.25 ± 3.31	18.55 ± 0.88
Donepezil	80.65 ± 0.81	69.66 ± 3.91	31.15 ± 3.79	25.15 ± 3.31	11.25 ± 3.21	37.92 ± 0.68
**8**	80.66 ± 0.81	43 ± 2.68	22.66 ± 1.79	14.16 ± 2.56	5.66 ± 1.97	42.20 ± 0.68
**9**	80.5 ± 0.84	40.83 ± 0.98	23.5 ± 2.92	15.83 ± 3.37	2.66 ± 0.68	48.55 ± 0.68
**10**	80.33 ± 1.09	36 ± 1.09	25.33 ± 1.79	17 ± 1.67	3.66 ± 1.88	46.45 ± 0.8
**15**	80.5 ± 0.86	49.83 ± 0.75	29.16 ± 3.07	11.5 ± 1.04	3.83 ± 1.34	46.77 ± 0.4
**16**	76 ± 3.3	30.8 ± 0.75	21 ± 0.81	16 ± 1.54	5.33 ± 1.24	42.48 ± 0.8
**17**	81 ± 0.89	32.83 ± 1.83	22.66 ± 1.79	12.33 ± 2.25	4.16 ± 0.74	44.48 ± 0.68
**18**	80.66 ± 0.81	44.83 ± 3.125	24.66 ± 1.24	13.83 ± 2.63	3.33 ± 1.37	46.89 ± 0.54
**19**	54.83 ± 1.47	44.83 ± 2.71	21.33 ± 2.68	13.16 ± 2.31	3.66 ± 0.94	46.52 ± 0.68
**23**	55.33 ± 1.96	45.16 ± 0.75	35.33 ± 2.56	11.83 ± 1.94	4.16 ± 1.067	44.75 ± 0.45

The values are shown as mean ± SD, p < 0.05 as compared to scopolamine. One-way ANOVA followed by Tukey’s test was employed to find out the intergroup variation and values are considered statistically significant (p < 0.0001).

*SD* standard deviation, *TSTQ* time spent in the target quadrant.

**Figure 2:**
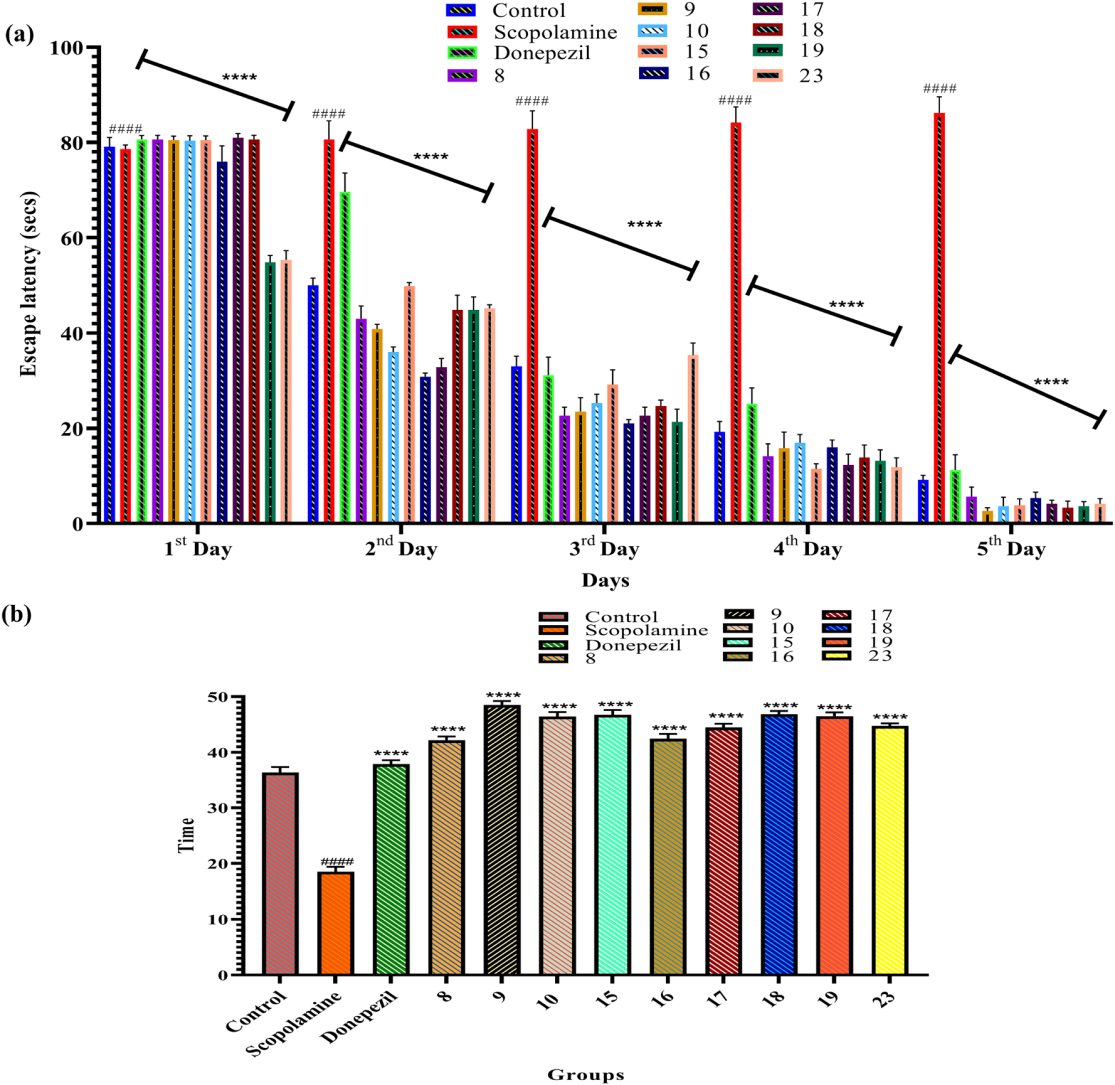
Behavioural study results: (**a**) Effect of compounds 8, 9, 10, 15, 16, 17, 18, 19 and 23 on escape latency compared to the scopolamine-treated group of mice. Values are expressed as Mean ± SD, n = 6. ^####^p < 0.0001 (positive control is compared with the normal control), ****p < 0.0001 (treatment groups are compared with positive control) (**b**) Effect of compounds 8, 9, 10, 15, 16, 17, 18, 19 and 23 on time spent compared to the scopolamine treated group of mice. Values are expressed as Mean ± SD, n = 6. ^####^p < 0.0001 (positive control is compared with the normal control), ****p < 0.0001 (treatment groups are compared with positive control).

The behavioural assessment for memory impairment in mice treated with scopolamine (1.5 mg/kg, i.p.) depicted an increase in escape latency in the Morris water maze task (from day 1 to 5) due to abnormality in learning and memory. Treatment with synthesized derivatives 8, 9, 10, 15, 16, 17, 18, 19 and 23 (2 mg/kg) resulted in significantly reduced escape latency. In probe trial (day 5), significantly decreased TSTQ in scopolamine-treated mice were appreciably reversed on treatment with newly synthesized compounds 8, 9, 10, 15, 16, 17, 18, 19 and 23. Compounds 9 and 18 emerged as the most potent compounds with significantly improved cognitive defects at 2 mg/kg, which is better than donepezil (2 mg/kg) and other compounds showed comparable effects with standard drugs. The effects of newly synthesized compounds on the behavioural pattern in mice treated with scopolamine have been represented in Fig. 2a,b.

#### Biochemical assays

In the present study, the levels of four biochemical markers were measured in THS of mice (scopolamine, i.p.) and treated with newly synthesized compounds 8, 9, 10, 15, 16, 17, 18, 19 and 23 along with standard drug donepezil as reported earlier^37,38^. The four markers were:

Acetylcholinesterase (AChE): an enzyme that breaks down acetylcholine, a neurotransmitter that is important for memory and learning.

Lipid peroxidation (LPO): a measure of damage to cell membranes caused by free radicals.

Nitrite: a measure of the production of nitric oxide, a molecule that can be both beneficial and harmful to the brain.

Oxidative stress: an imbalance between free radicals and antioxidants.

**Figure 3:**
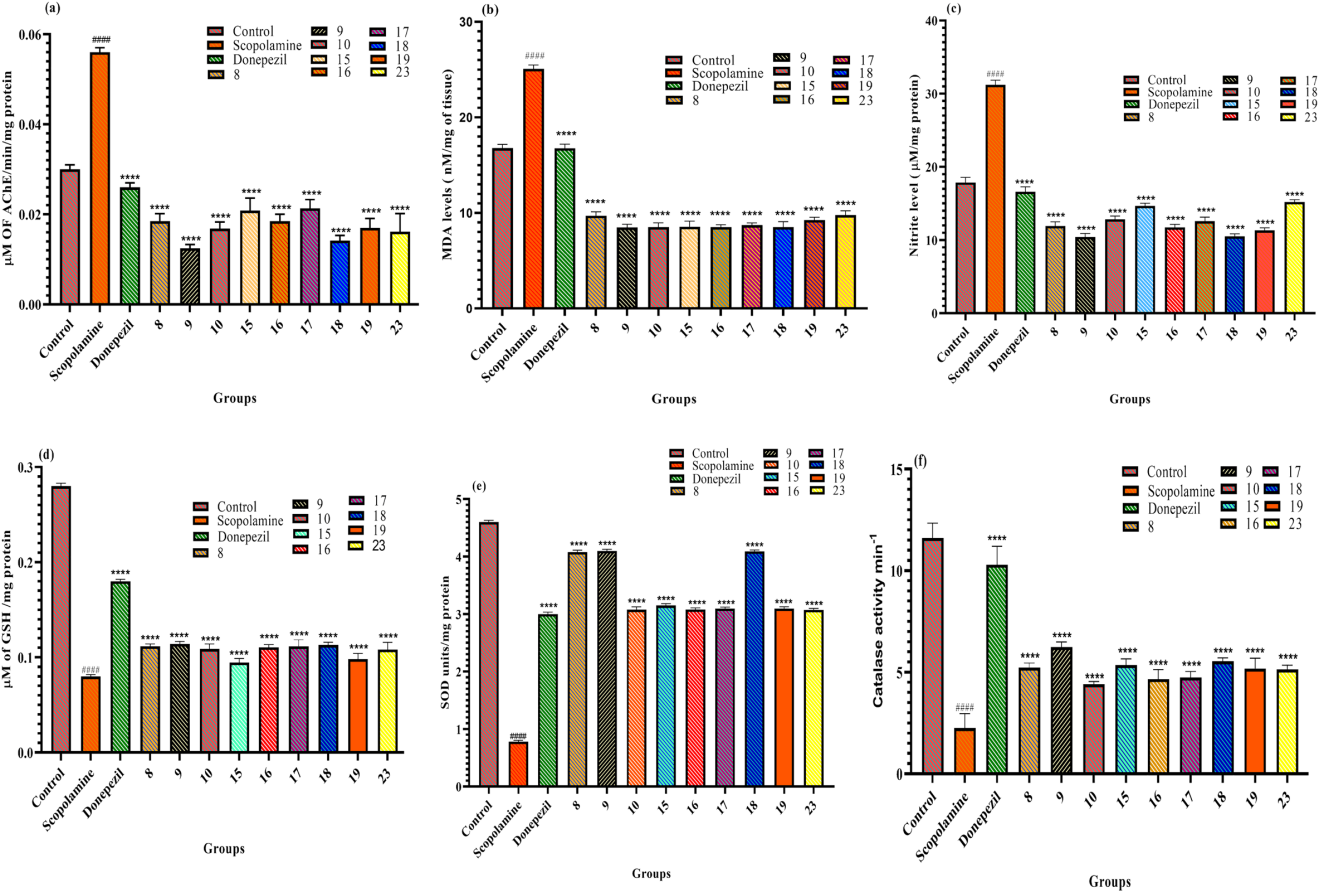
(**a–f**) Effect of compounds 8, 9, 10, 15, 16, 17, 18, 19 and 23 on acetylcholinesterase level (**a**), lipid peroxidation (**b**), nitrite level (**c**) and antioxidant profile (**d**–**f**) compared to the scopolamine treated group of mice. Values are expressed as Mean ± SD, n = 6. ^####^p < 0.0001 (positive control is compared with the normal control), ****p < 0.0001 (treatment groups are compared with positive control).

This study suggests that the newly synthesized compounds were effective as anti-AD agents. Of the nine compounds tested, compounds 9 and 18 were the most effective in restoring normal levels of the biochemical markers and inhibiting the production of reactive oxygen and nitrogen species (ROS and RNS), which are molecules that play a role in neuroinflammation. Detailed levels of the measured biomarkers (mean ± SEM) have been shown in Table 2. The biochemical estimation of the measured biomarkers mice THS on treatment with compounds 8, 9, 10, 15, 16, 17, 18, 19 and 23 has been illustrated in Fig. 3a–f. To better understand how these compounds bind to AChE and how different functional groups might affect AChE inhibition, various *in-silico* studies were performed.

**Table 2:** Escape latency data of compounds 8, 9, 10, 15, 16, 17, 18, 19 and 23.

Compound	μM of AChE/min/mg protein	LPO assay, MDA levels (nM/mg of tissue)	Nitrite assay, nitrite conc. (μM/mg) protein	GSH assay, μmol of GSH/mg Protein	SOD assay, SOD units/mg protein	Catalase assay, Catalase activity min^−1^
Control	0.03 ± 0.01	16.78 ± 0.04	17.88 ± 0.06	0.28 ± 0.001	4.6 ± 0.03	11.61 ± 0.07
Scopolamine	0.05 ± 0.056	25.09 ± 0.03	31.21 ± 0.06	0.08 ± 0.002	0.78 ± 0.01	2.25 ± 0.031
Donepezil	0.02 ± 0.01	16.76 ± 0.04	16.63 ± 0.06	0.18 ± 0.001	3.0 ± 0.03	10.3 ± 0.09
**8**	0.01 ± 0.01	9.71 ± 0.03	11.92 ± 0.05	0.11 ± 0.001	4.07 ± 0.03	5.23 ± 0.02
**9**	0.01 ± 0.01	8.48 ± 0.02	10.42 ± 0.04	0.11 ± 0.002	4.09 ± 0.02	6.23 ± 0.02
**10**	0.01 ± 0.01	8.52 ± 0.05	12.82 ± 0.04	0.10 ± 0.005	3.07 ± 0.04	4.3 ± 0.01
**15**	0.02 ± 0.02	8.54 ± 0.02	14.66 ± 0.04	0.09 ± 0.003	3.14 ± 0.03	5.35 ± 0.03
**16**	0.01 ± 0.01	8.52 ± 0.05	11.74 ± 0.03	0.11 ± 0.002	3.07 ± 0.02	4.66 ± 0.04
**17**	0.02 ± 0.01	8.73 ± 0.05	12.57 ± 0.05	0.11 ± 0.006	3.09 ± 0.02	4.76 ± 0.02
**18**	0.01 ± 0.01	8.52 ± 0.02	10.51 ± 0.03	0.11 ± 0.001	4.08 ± 0.02	5.53 ± 0.01
**19**	0.01 ± 0.02	9.25 ± 0.02	11.31 ± 0.03	0.09 ± 0.005	3.09 ± 0.03	5.17 ± 0.05
**23**	0.01 ± 0.04	9.77 ± 0.04	15.2 ± 0.03	0.10 ± 0.007	3.07 ± 0.02	5.13 ± 0.03

The values are shown as mean ± SD, p < 0.05 as compared to scopolamine. One-way ANOVA followed by Tukey’s test was employed to find out the intergroup variation and values are considered statistically significant (p < 0.0001).

*AChE* acetylcholinesterase, *GSH* reduced glutathione, *LPO* lipid peroxidation; *MDA* malondialdehyde, *SD* standard deviation, *SOD* superoxide dismutase.

### In-silico studies

#### Molecular docking studies

All the synthesized compounds underwent docking in XP mode, targeting the active site of human acetylcholinesterase bound with donepezil (PDB-4EY7). A receptor grid was established at the donepezil binding site, which was originally co-crystallized with the protein (PDB ID 4EY7). To verify the docking procedure's accuracy, we conducted a test by removing donepezil from the protein, redocking it, and computing the Root Mean Square Deviation (RMSD) value. The RMSD value was determined to be 0.6083, confirming the validity of the docking protocol.

The XP docking results yielded several parameters, with significant outputs including the docking score, glide score, and glide emodel, which are presented in Supplementary Table S2. After docking, the binding free energy of the protein–ligand complexes was computed using Prime, and these results are presented as MM-GBSA dG bind in Table 3. For donepezil, the docking score and MM-GBSA dG bind value were calculated as − 16.126 and − 86.39 kcal/mol, respectively (Table 3). The docking score and interactions with amino acid residues within the active site of donepezil were established as the reference, and the outcomes for the synthesized compounds were compared to these standards. Donepezil exhibited hydrogen bond interactions with the PHE295 residue. Additionally, the protonated nitrogen of the piperidinyl group engaged in pi-cation interactions with TRP86, TYR337, and PHE338 residues, as well as pi-pi interactions with TRP86 and TRP286 residues. Hydrophobic interactions were observed with TYR72, TRP86, TYR124, TYR133, TRP286, LEU289, VAL294, PHE295, FHE297, TYR341, TYR337, PHE338, and ILE451 residues. Further details regarding the interaction between donepezil and AChE can be found in Supplementary Fig. S2 online. In comparison to donepezil, compound 9 displayed similar interactions, including pi–pi interaction with TRP86, TYR337, and TRP86, as illustrated in Supplementary Fig. S3 online.

**Table 3:** Statistical values of different parameters of the built atom-based 3D-QSAR model.

# Factors	SD	R^2^	R^2^ CV	R^2^ Scramble	Stability	F	P	RMSE	Q^2^	Pearson-r
1	0.6837	0.5597	0.0736	0.6128	0.718	58.5	9.84E−10	0.54	0.6251	0.8323
2	0.3542	0.8844	0.2262	0.844	0.44	172.2	8.24E−22	0.44	0.7507	0.8752
3	0.1966	0.9652	0.3027	0.925	0.393	406.4	4.43E−32	0.49	0.6808	0.8252
4	0.1183	0.9877	0.2879	0.959	0.322	861.1	2.01E−40	0.41	0.7762	0.8918
5	0.0965	0.992	0.3058	0.9714	0.33	1040.2	7.40E−43	0.42	0.7675	0.8888

*QSAR* quantitative structure–activity relationship, *RMSE* root mean square error, *SD* standard deviation.

The molecular docking results suggest that compound 9 is a promising candidate for the development of new drugs to treat Alzheimer's disease. Compound 9 showed similar interactions with the acetylcholinesterase enzyme as donepezil, a known inhibitor of acetylcholinesterase. In particular, both compounds formed pi-pi interactions with the amino acid residues TRP86, TYR337, and TRP86. Pi-pi interactions are a type of stacking interaction that occurs between two aromatic rings. These interactions are important for stabilizing protein–ligand complexes and are often targeted by drug designers. The fact that compound 9 forms similar pi-pi interactions with acetylcholinesterase as donepezil suggests that it may be an effective inhibitor of the enzyme. This is further supported by the high docking score and binding free energy calculated for compound 9.

#### MD simulation study

Different biological assays suggested that compound 9 is a potent AChE inhibitor with anti-AD potential. To further understand the interaction of compound 9 with AChE, molecular docking was conducted which suggested that compound 9 might show similar non-bonding interactions like donepezil. However, the ligand-interaction diagram (generated after XP-docking) showed that compound 9 didn’t form any H-bond with AChE. Hence, to explore the interaction of compound 9 with AChE, an MD simulation study was performed for 100 ns. Analysis of MD simulation results gave key insights regarding the conformational changes in the protein residues and ligands and their impact on the stability of the protein–ligand complex. These conclusions were drawn based on several plots like RMSD, H-bond plot, SASA (solvent accessible surface area), and Rg (radius of gyration).

**Figure 4:**
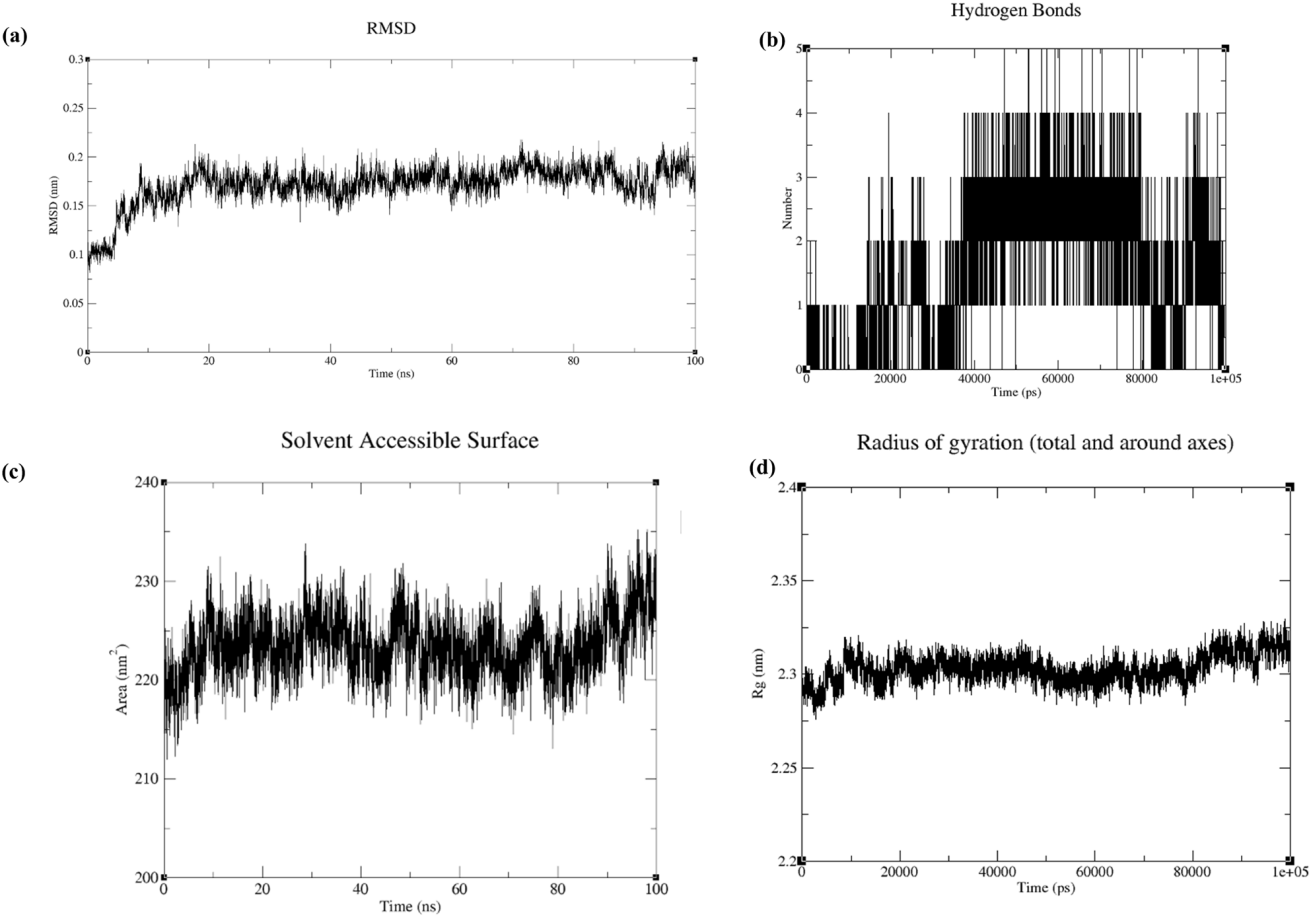
MD study results for compound 9 and AChE complex. (**a**) RMSD plot (**b**) H-bond plot (**c**) SASA plot (d) Rg plot.

RMSD plot of compound 9 and AChE (Fig. 4a), showed that the system attained equilibration at around 10 ns. The complex showed stability throughout the simulation period post-equilibration as the average RMSD deviation was between 0.1 and 0.2 nm. Intermolecular H-bond interactions play a crucial role in the stability of ligand-receptor complexes and hence are considered important in the potency of a molecule. The number and distribution of H-bonds between compound 9 and AChE have been shown in Fig. 4b. Throughout the simulation period, the number of H-bonds formed ranged between one to five but mostly clustered around two to three. It suggested that compound 9 might form H-bond with AChE which we could not observe during docking studies. SASA plots represent the change in the folding pattern of the inner hydrophobic core to the outer hydrophilic surface of the protein structure due to the interaction with the ligand. As shown in Fig. 4c, the average deviation of the accessible surface area was in the range of 210 to 235 nm^2^_._ It suggested that there was no abrupt change in the folding pattern of AChE due to the interaction with compound 9 and hence, the complex might be stable. Rg plots (Fig. 4d) also suggested that AChE didn’t lose its compactness after forming a complex with compound 9 as reflected by the Rg value for this complex.

#### Atom-based 3D-QSAR study

Based on the 60 ligands with substantial structural diversity, an atom-based 3D-QSAR model was built. A total of 48 ligands were selected as the training set and 12 as the test set (see Supplementary Table S1 online). As shown in Table 3, for PLS factor 5, the developed model was both robust and reliable. Q^2^ value depicting the predictive ability of the model for the training set was found to be 0.992 while Q^2^ was 0.7675. The standard deviation was also found to be 0.0965 with an R^2^ scramble value of 0.9714. The predictive ability of the model is also shown in Supplementary Fig. S4a (training set) and Fig. S4b (test set). The model predicted the activity of all the compounds with comparable accuracy to the experimental value. The predicted pIC_50_ values for compounds 8, 9, 10, 15, 16, 17, 18, 19, and 23 were 8.06398, 7.99922, 7.94539, 7.62048, 7.73745, 7.64993, 7.965, 8.04471, and 7.41003 respectively as shown in Supplementary Table S1.

Based on this result, the atomic contribution was computed for the AChE inhibitory ability of the ligands. As shown in Supplementary Table S3, atoms contributing towards the hydrophobic or non-polar nature of the ligands were majorly responsible for the AChE inhibitory activity. The hydrophobic atoms’ contribution was found to be 0.626. Atoms contributing towards electron withdrawing activity (0.037) and H-bond donating ability (0.032) were other major contributors. Detailed contribution of different atoms for compound 9 has been shown in Supplementary Fig. S5a–d. Considering these insights regarding atom-type contribution, if we analyze the compounds of the present study, most of the compounds possess these chemical features. The presence of chloro or floro groups remarkedly enhanced the activity of these compounds apart from multiple rings contributing towards hydrophobicity.

#### ADME prediction

The drug-likeness of these molecules was predicted using the QikProp module of Maestro. As shown in Table 4, all molecules showed favourable predictions regarding their H-bond donor/acceptor ability, bioavailability, solubility, PSA (polar surface area), or central nervous system (CNS) availability. The compounds were also found to be non-cardiotoxic as shown by their predicted QPlogHERG values.

**Table 4:** ADME predictions of the synthesized compounds.

Title	CNS	MW	donorHB	accptHB	QPlogPo/w	QPlogHERG	% human oral absorption	PSA
8	1	491.576	1	11	2.219	− 5.157	77.629	97.032
9	1	509.567	1	11	2.374	− 5.152	65.904	97.204
10	2	396.916	0	4.5	5.365	− 6.949	100	30.328
15	2	410.942	0	4.5	5.7	− 7.073	100	31.081
16	2	424.969	0	4.5	6.108	− 7.322	100	32.069
17	2	468.594	0	5.25	6.574	− 7.89	100	38.646
18	2	506.567	0	4.5	7.523	− 7.944	100	30.388
19	1	363.458	2	7	2.324	− 5.526	80.06	81.691

*ADME* absorption, distribution, metabolism, excretion; *accptHB* hydrogen-bond acceptor; *CNS* central nervous system; *donorHB* hydrogen-bond donor; *MW* molecular weight; *PSA* polar surface area; *QPlogPo/w* predicted octanol/water partition coefficient.

## Conclusions

In summary, the newly synthesized derivatives possess neuroprotective activity for the management of Alzheimer’s disease. All the synthesized compounds, 8, 9, 10, 15, 16, 17, 18, 19 and 23 prevent the progression of scopolamine-induced cognitive defects in the MWM animal model and substantial inhibition of scopolamine encouraged acetylcholinesterase activity, oxidative stress and nitrosative-stress were perceived. Molecular modelling studies also suggested that these compounds have the required chemical features to bind with AChE and lead to its inhibition. In conclusion, among all synthesized compounds 9 and 18 showed the highest potency with better neuroprotection compared to standard drug donepezil. The study indicated that the synthesized derivatives if explored extensively, may act as lead candidates for the management of neurodegenerative disorders.

### Supplementary Information

Supplementary Information.

## Data Availability

Spectral data and QSAR model input data are provided in the supplementary file. Any other relevant data will be shared on reasonable request. The corresponding author should be contacted if someone wants to request the data from this study.
