# Supplementary material for: Investigation, scaffold hopping of novel donepezil-based compounds as anti-Alzhiemer’s agents: synthesis, in-silico and pharmacological evaluations
Source: Sci Rep. 2024 Jan 19;14:1687. doi: 10.1038/s41598-024-51713-4 (PMC10799042; doi:10.1038/s41598-024-51713-4)
Supplement: Supplementary file 1 — Supplementary Information. [file 41598_2024_51713_MOESM1_ESM.docx]

**Table S1.** Ligands used to build atom-based 3D-QSAR model.

| Sr No. | Structure | IC50 (µM) | pIC50 | Predicted Activity^5#^ | QSAR Set |
| --- | --- | --- | --- | --- | --- |
| 1 |  | 0.01 | 8 | 8.06398 | training |
| 2 |  | 0.01 | 8 | 7.99922 | training |
| 3 |  | 0.01 | 8 | 7.94539 | training |
| 4 |  | 0.02 | 7.698 | 7.62048 | training |
| 5 |  | 0.02 | 7.698 | 7.73745 | training |
| 6 |  | 0.01 | 8 | 7.64993 | test |
| 7 |  | 0.01 | 8 | 7.965 | training |
| 8 |  | 0.01 | 8 | 8.04471 | training |
| 9 |  | 0.01 | 8 | 7.41003 | test |
| 10 |  | 0.02 | 7.698 | 7.69582 | training |
| 11 |  | 0.021 | 7.677 | 7.68039 | training |
| 12 |  | 0.018 | 7.744 | 7.76021 | training |
| 13 |  | 0.031 | 7.508 | 7.57701 | training |
| 14 |  | 0.033 | 7.481 | 7.47479 | training |
| 15 |  | 0.022 | 7.657 | 7.63978 | training |
| 16 |  | 0.022 | 7.657 | 7.62639 | training |
| 17 |  | 0.02 | 7.698 | 7.14356 | test |
| 18 |  | 0.028 | 7.552 | 7.54925 | training |
| 19 |  | 0.024 | 7.619 | 7.11666 | test |
| 20 |  | 0.029 | 7.537 | 7.49424 | training |
| 21 |  | 0.026 | 7.585 | 7.57198 | training |
| 22 |  | 0.022 | 7.657 | 7.64554 | training |
| 23 |  | 0.032 | 7.494 | 6.90264 | test |
| 24 |  | 0.022 | 7.657 | 7.62433 | training |
| 25 |  | 0.026 | 7.585 | 7.59545 | training |
| 26 |  | 0.054 | 7.267 | 7.1272 | training |
| 27 |  | 0.102 | 6.991 | 7.03315 | training |
| 28 |  | 1.263 | 5.898 | 6.07613 | training |
| 29 |  | 0.093 | 7.031 | 7.06098 | training |
| 30 |  | 0.09 | 7.045 | 7.13554 | training |
| 31 |  | 0.153 | 6.815 | 6.84613 | training |
| 32 |  | 1.45 | 5.838 | 5.98601 | training |
| 33 |  | 0.135 | 6.869 | 7.32123 | test |
| 34 |  | 0.051 | 7.292 | 7.33838 | training |
| 35 |  | 0.09 | 7.045 | 7.03365 | training |
| 36 |  | 0.02 | 7.698 | 7.67656 | training |
| 37 |  | 0.012 | 7.92 | 7.91211 | training |
| 38 |  | 0.092 | 7.036 | 7.43082 | test |
| 39 |  | 0.05 | 7.301 | 7.25511 | training |
| 40 |  | 76.6 | 4.115 | 4.02132 | training |
| 41 |  | 80.7 | 4.093 | 4.12072 | training |
| 42 |  | 9.95 | 5.002 | 5.00457 | test |
| 43 |  | 23.6 | 4.627 | 4.53798 | training |
| 44 |  | 1.51 | 5.821 | 6.37497 | test |
| 45 |  | 1.86 | 5.73 | 5.66626 | training |
| 46 |  | 8.23 | 5.084 | 5.21304 | training |
| 47 |  | 0.298 | 6.525 | 6.59317 | training |
| 48 |  | 0.123 | 6.91 | 6.76488 | training |
| 49 |  | 0.662 | 6.179 | 6.13653 | training |
| 50 |  | 1.45 | 5.838 | 5.97626 | training |
| 51 |  | 0.378 | 6.422 | 6.29748 | training |
| 52 |  | 0.174 | 6.759 | 6.65229 | test |
| 53 |  | 0.791 | 6.101 | 6.01459 | training |
| 54 |  | 0.34 | 6.468 | 6.42404 | test |
| 55 |  | 0.22 | 6.657 | 6.50955 | training |
| 56 |  | 0.502 | 6.299 | 6.50145 | training |
| 57 |  | 0.434 | 6.362 | 6.64132 | test |
| 58 |  | 0.269 | 6.57 | 6.34762 | training |
| 59 |  | 0.768 | 6.114 | 6.34259 | training |
| 60 |  | 1.55 | 5.809 | 5.76921 | training |

^#^Predicted activity has been shown for PLS (partial least square) factor 5.

**Table S2.** Docking result of donepezil and compound 9.

| **S.NO** | **Compound** | **XP G score (Kcal/mol)** | **MM-GBSA dG bind (Kcal/mol)** | **Interacting residues** |
| --- | --- | --- | --- | --- |
| 1 | Donepezil | -16.126 | -86.39 | PHE295 (H-bond, 2.09), TRP286 (pi-pi), TYR337 (pi cation), PHE338 (pi cation), TRP86 (pi-pi, pi cation) |
| 2 | 9 | -6.288 | -43.06 | TRP286 (pi-pi), TYR337 (pi-pi), TRP86 (pi-pi) |

**Table S3.** Atom-type contribution towards AChE inhibition of the ligands.

| # Factors | H-bond donor | Hydrophobic/non-polar | Negative ionic | Positive ionic | Electron-withdrawing |
| --- | --- | --- | --- | --- | --- |
| 1 | 0.026 | 0.654 | 0.031 | 0.017 | 0.246 |
| 2 | 0.029 | 0.63 | 0.033 | 0.019 | 0.258 |
| 3 | 0.031 | 0.628 | 0.034 | 0.019 | 0.259 |
| 4 | 0.031 | 0.629 | 0.035 | 0.02 | 0.256 |
| 5 | 0.032 | 0.626 | 0.037 | 0.021 | 0.257 |

******

**Fig. S1.** Design strategy employed to get novel donepezil-based derivatives.


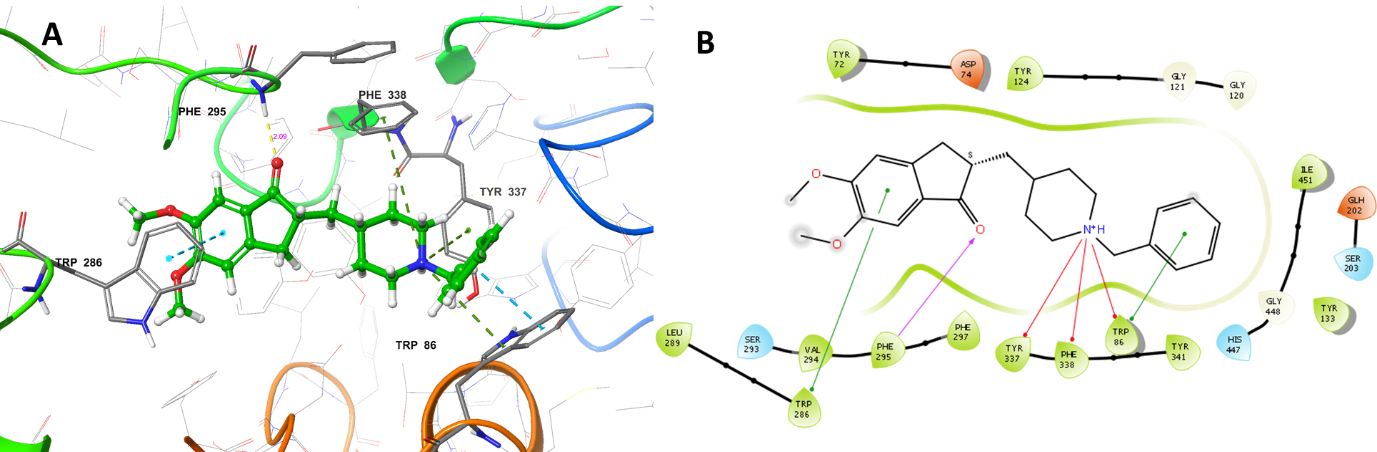


**Fig. S2.** Ligand interaction diagrams. (a) 3D interaction diagram of Donepezil (b) 2D interaction diagram of Donepezil


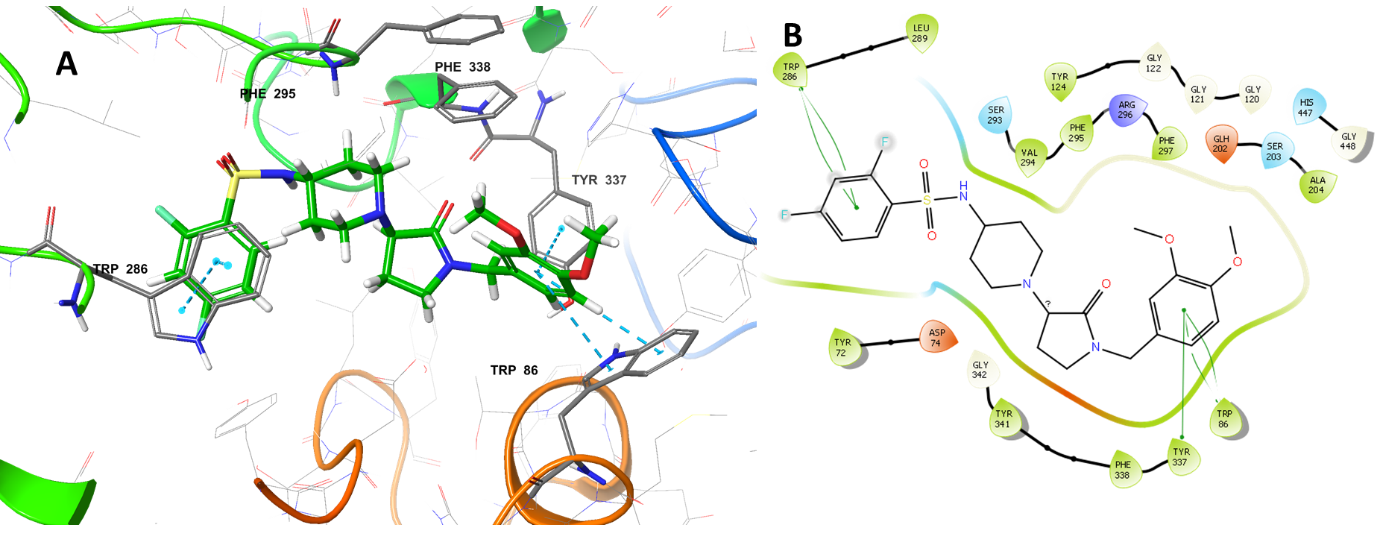


**Fig. S3.** Ligand interaction diagrams. 3D interaction diagram of compound 9 (d) 2D interaction diagram of compound 9.


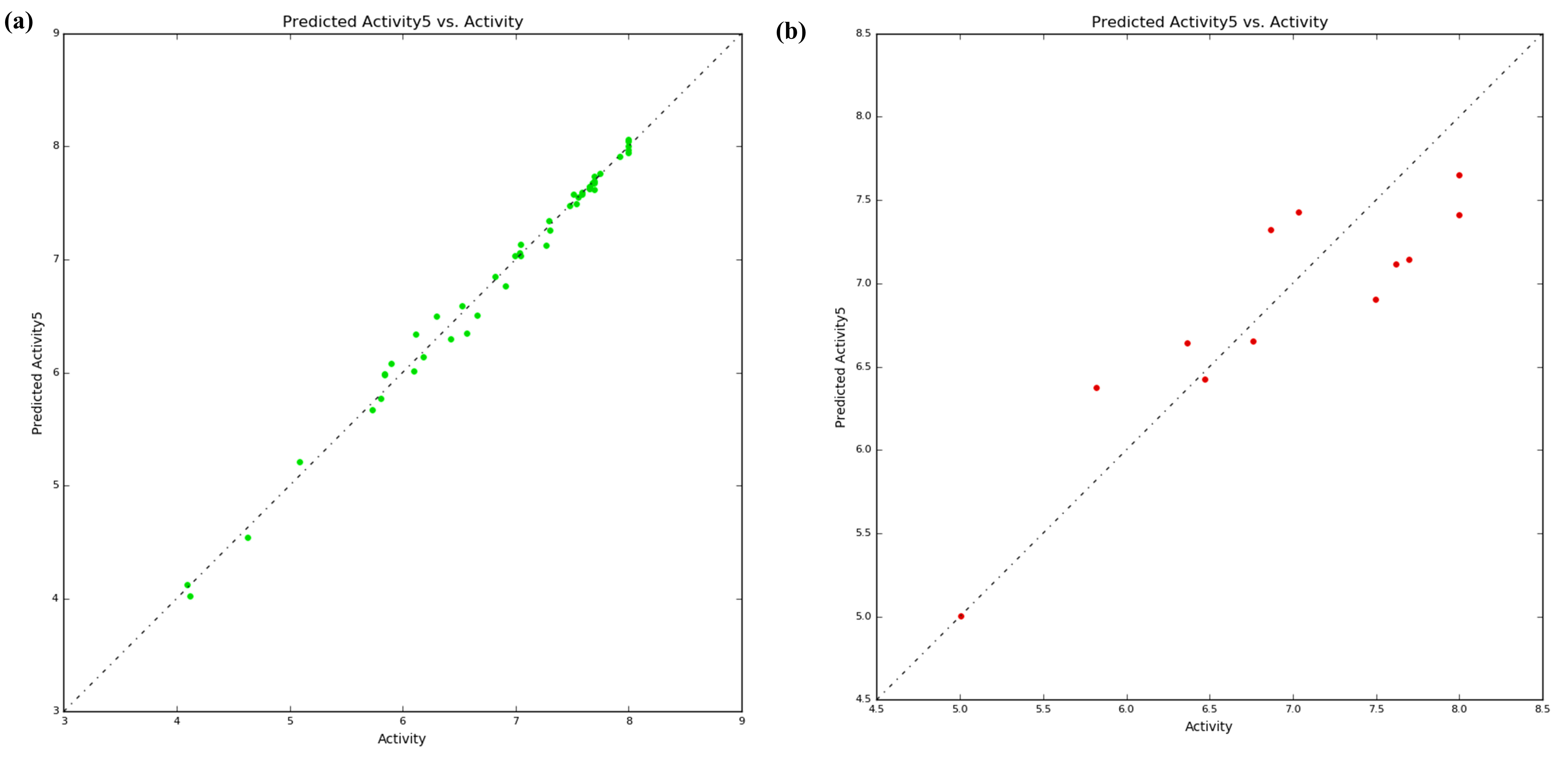


**Fig. S4.** Linear plot of actual activity vs. predicted activity for PLS factor 5. (a) Training set (b) Test set.


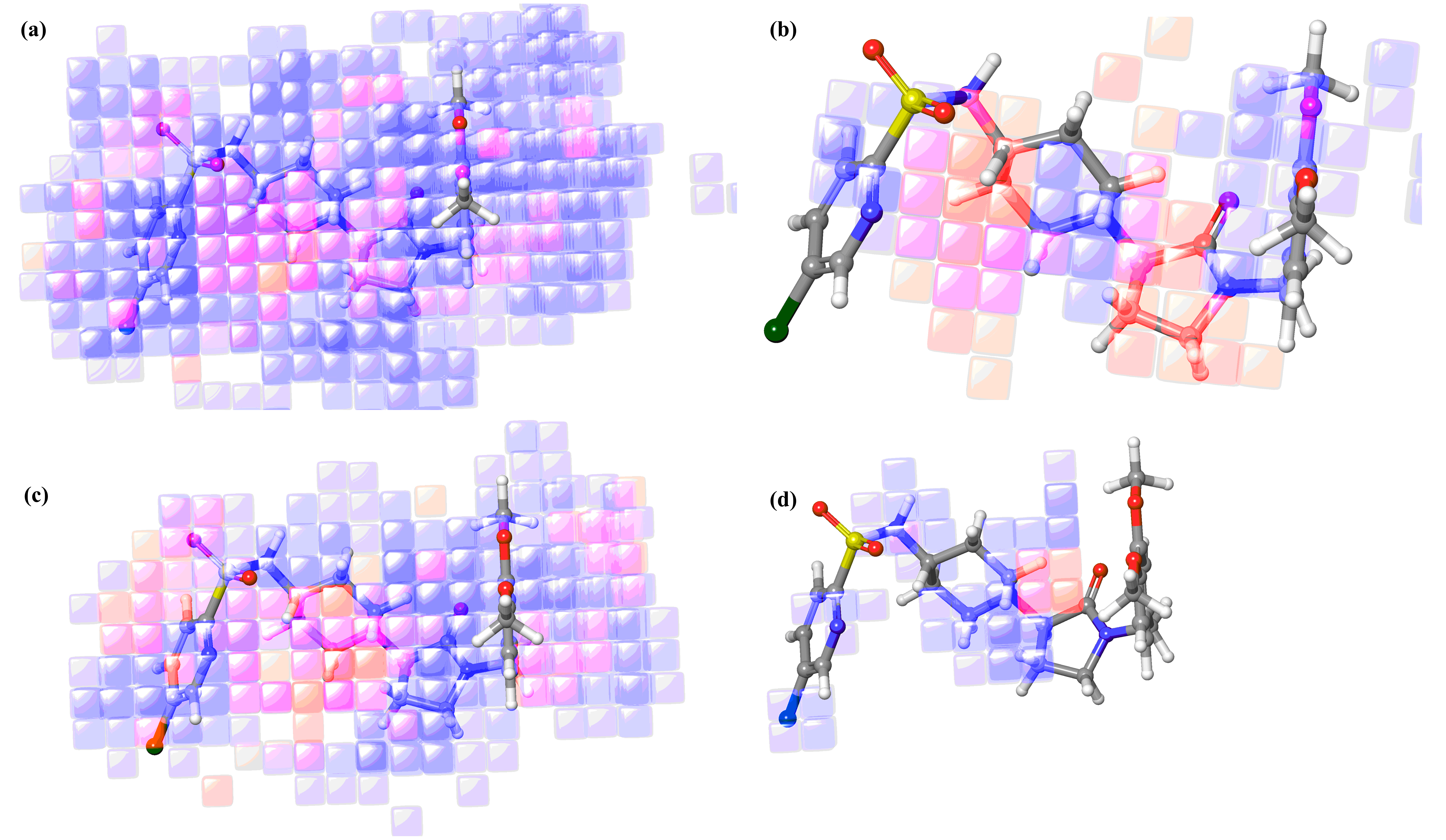


**Fig. S5.** Atom type contribution for compound 9. (a) Combined effect (b) Electron withdrawing effects (c) Hydrophobic/non-polar (d) H-bond donor effect.

**^1^H-NMR spectrum of compound-8 in DMSO-d_6_**

**
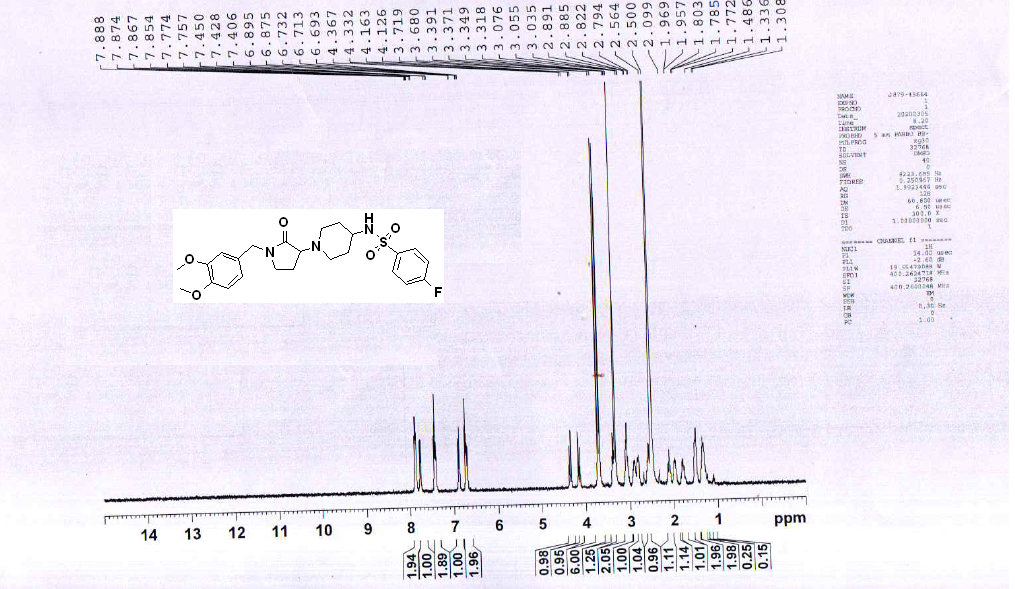
**

**Mass spectrum of Compound-8
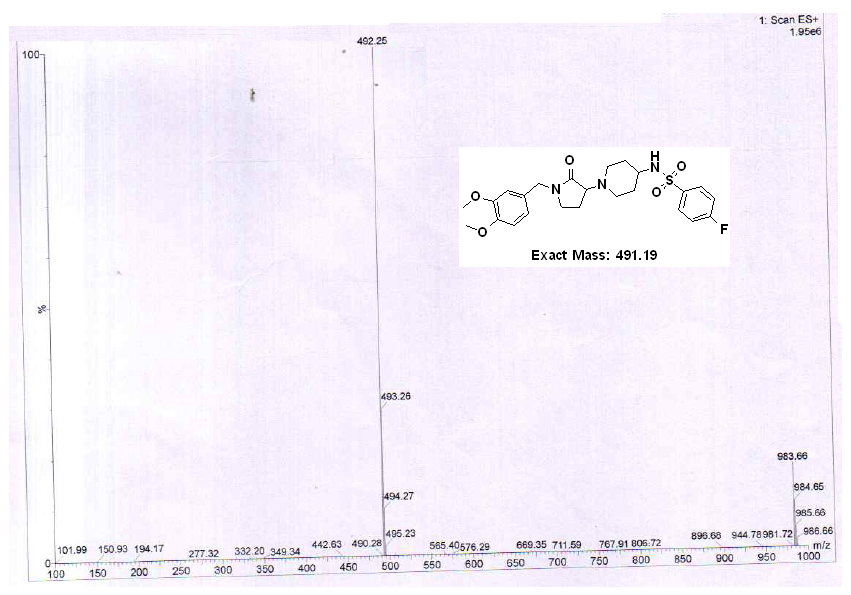
**

| **Calculated m/z** | 491.19 | **Ion formula** | C_24_H_30_FN_3_O_5_S | **Species** | [M+H]^+^ |
| --- | --- | --- | --- | --- | --- |
| **Result type (ESI mode)** | | | Target m/z | | |
| Positive ions | | | 492.25 | | |

**^1^H-NMR spectrum of compound-9 in DMSO-d_6_**

**
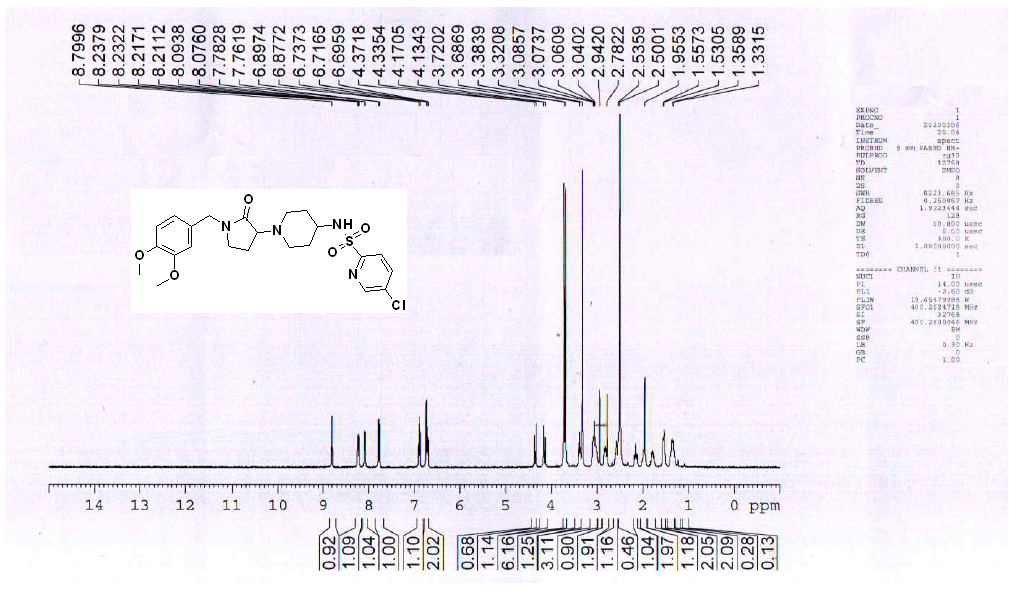
**

**Mass spectrum of Compound-9**

**
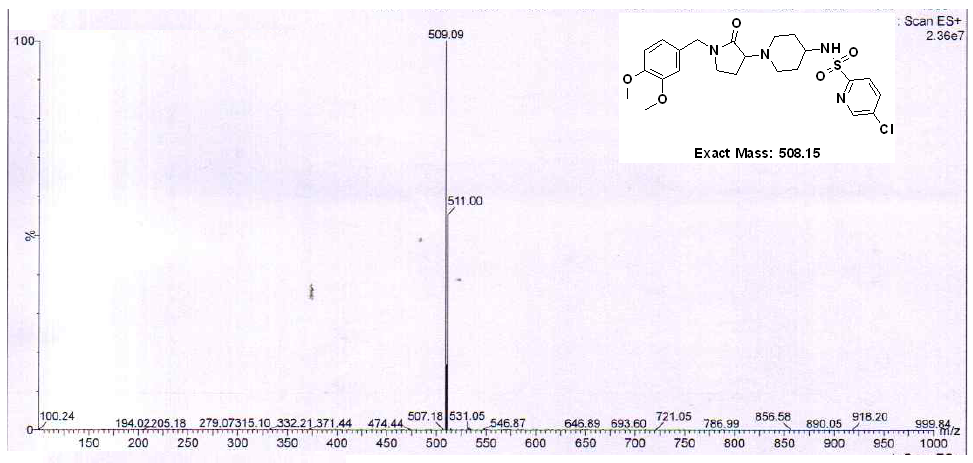
**

| **Calculated m/z** | 508.15 | **Ion formula** | C_23_H_29_ClN_4_O_5_S | **Species** | [M+H]^+^ |
| --- | --- | --- | --- | --- | --- |
| **Result type (ESI mode)** | | | Target m/z | | |
| Positive ions | | | 509.09 | | |

**^1^H-NMR spectrum of compound-10 in DMSO-d_6_**

**
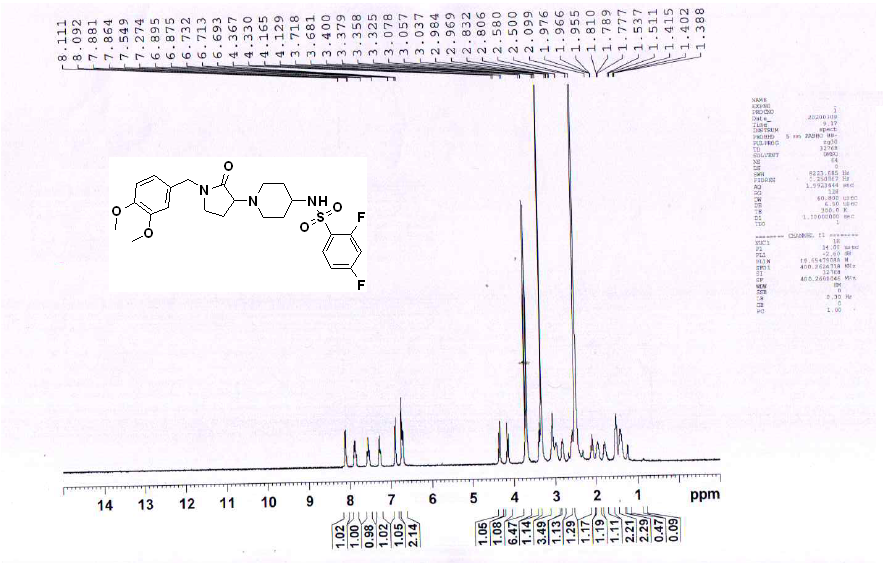
**

**Mass spectrum of Compound-10**

**
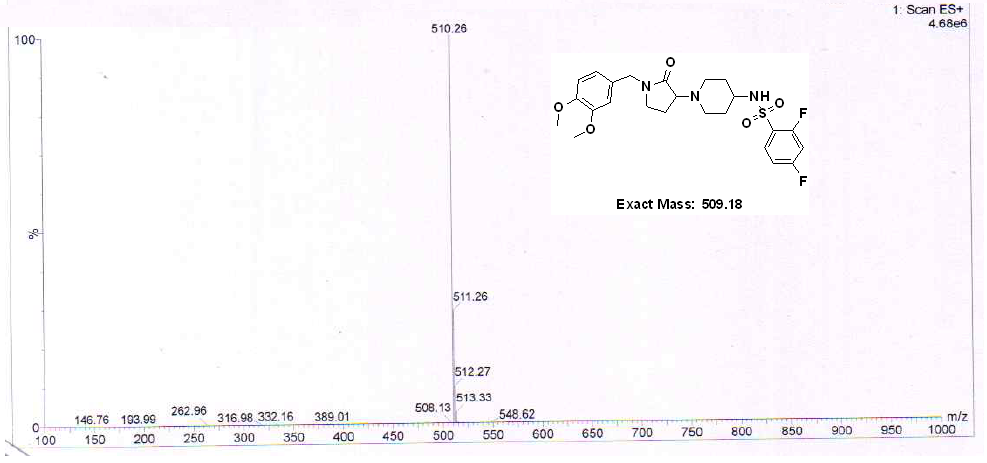
**

| **Calculated m/z** | 509.18 | **Ion formula** | C_24_H_29_F_2_N_3_O_5_S | **Species** | [M+H]^+^ |
| --- | --- | --- | --- | --- | --- |
| **Result type (ESI mode)** | | | Target m/z | | |
| Positive ions | | | 510.26 | | |

**^1^H-NMR spectrum of compound-15 in DMSO-d_6_**

**
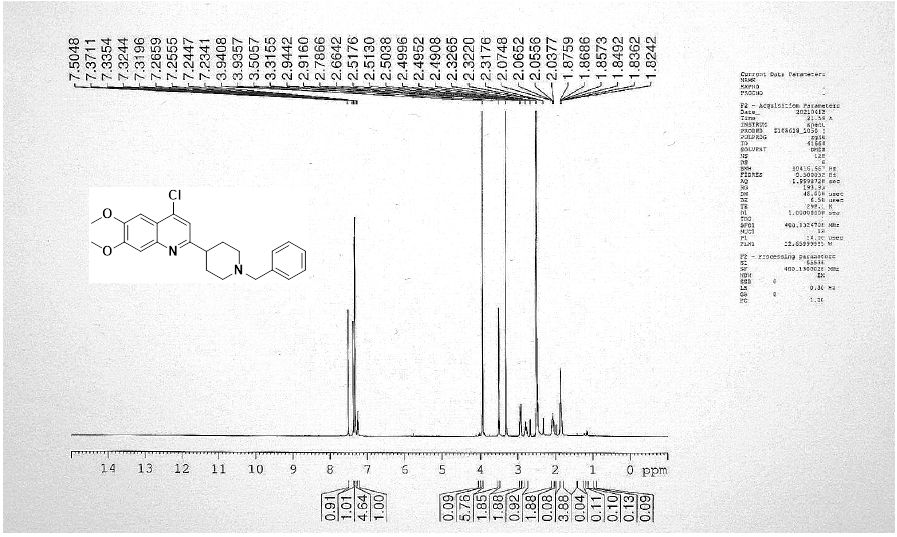
**

**Mass spectrum of Compound-15**

**
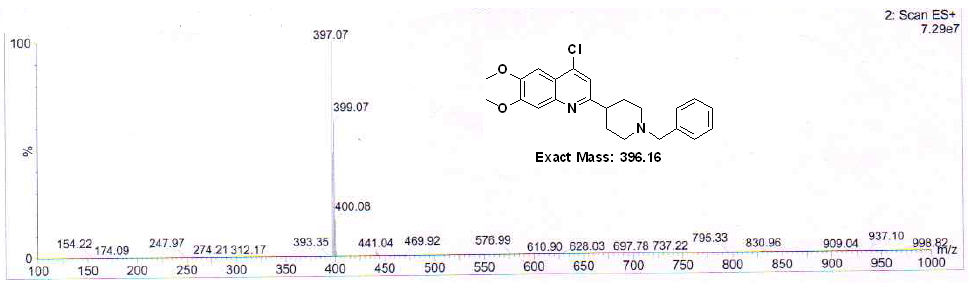
**

| **Calculated m/z** | 396.16 | **Ion formula** | C_23_H_25_ClN_2_O_2_ | **Species** | [M+H]^+^ |
| --- | --- | --- | --- | --- | --- |
| **Result type (ESI mode)** | | | Target m/z | | |
| Positive ions | | | 397.07 | | |

**^1^H-NMR spectrum of compound 16 in DMSO-d_6_**

**
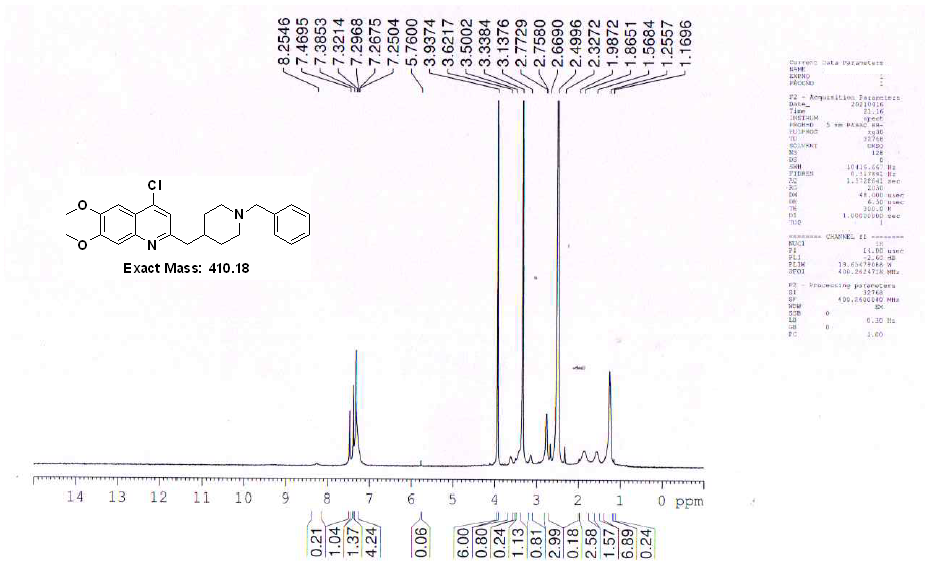
**

**Mass spectrum of Compound-16**

**
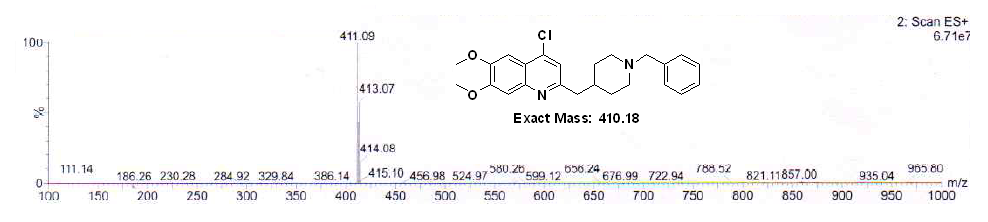
**

| **Calculated m/z** | 410.18 | **Ion formula** | C_24_H_27_ClN_2_O_2_ | **Species** | [M+H]^+^ |
| --- | --- | --- | --- | --- | --- |
| **Result type (ESI mode)** | | | Target m/z | | |
| Positive ions | | | 411.09 | | |

**^1^H-NMR spectrum of compound-17 in DMSO-d_6_**


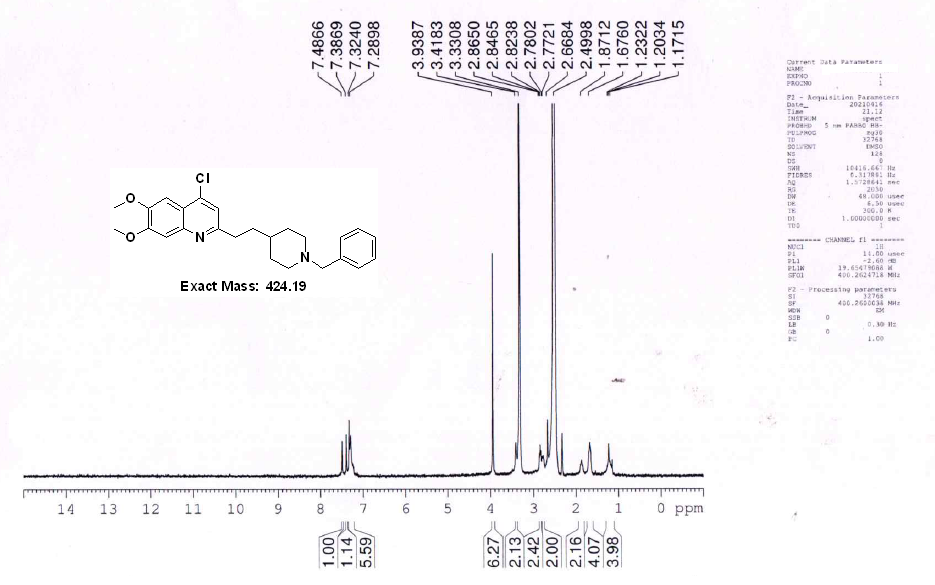


**Mass spectrum of Compound-17**


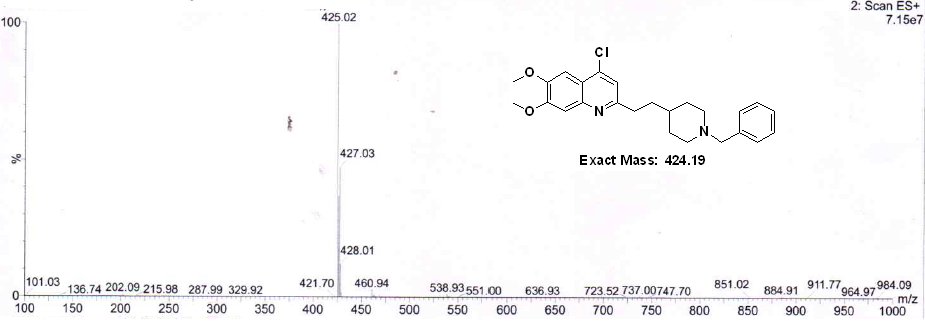


| **Calculated m/z** | 424.19 | **Ion formula** | C_25_H_29_ClN_2_O_2_ | **Species** | [M+H]^+^ |
| --- | --- | --- | --- | --- | --- |
| **Result type (ESI mode)** | | | Target m/z | | |
| Positive ions | | | 425.02 | | |

**^1^H-NMR spectrum of compound-18 in DMSO-d_6_**

**
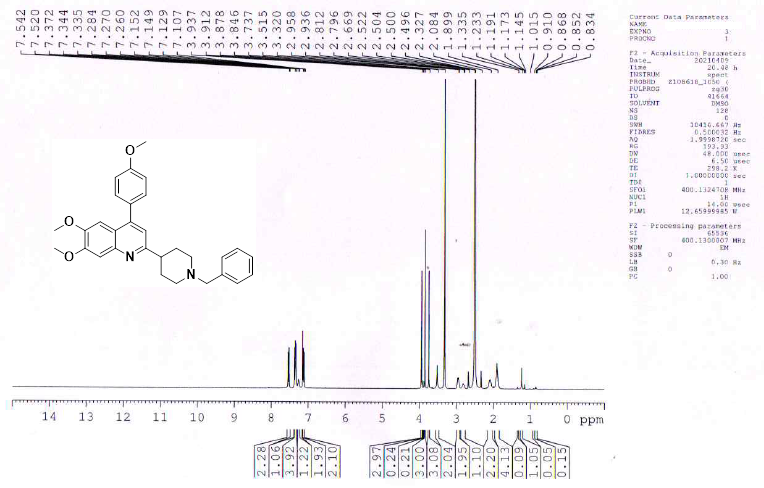
**

**Mass spectrum of Compound-18**

**
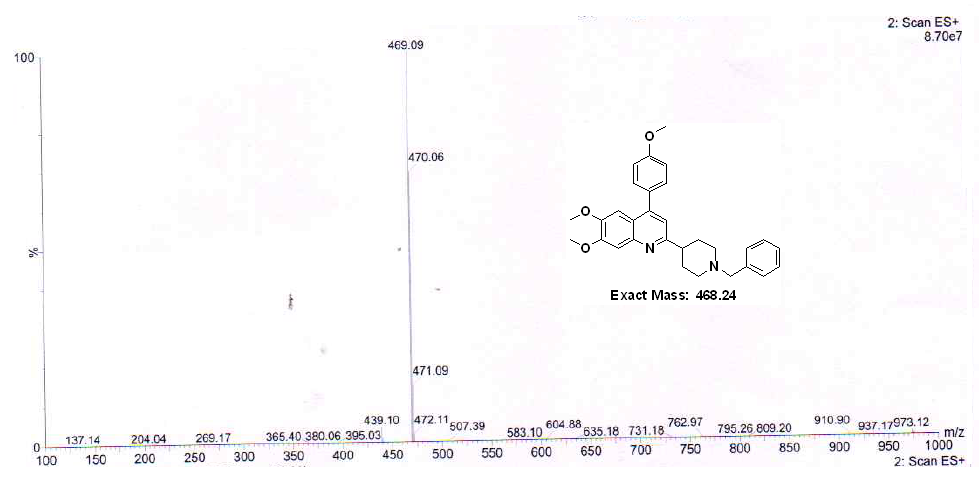
**

| **Calculated m/z** | 468.24 | **Ion formula** | C_30_H_32_N_2_O_3_ | **Species** | [M+H]^+^ |
| --- | --- | --- | --- | --- | --- |
| **Result type (ESI mode)** | | | Target m/z | | |
| Positive ions | | | 469.09 | | |

**^1^H-NMR spectrum of compound-19 in DMSO-d_6_
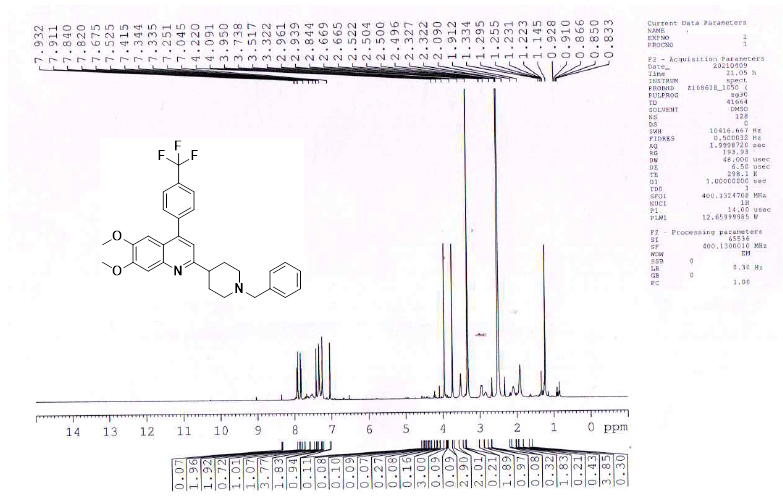
**

**Mass spectrum of Compound-19
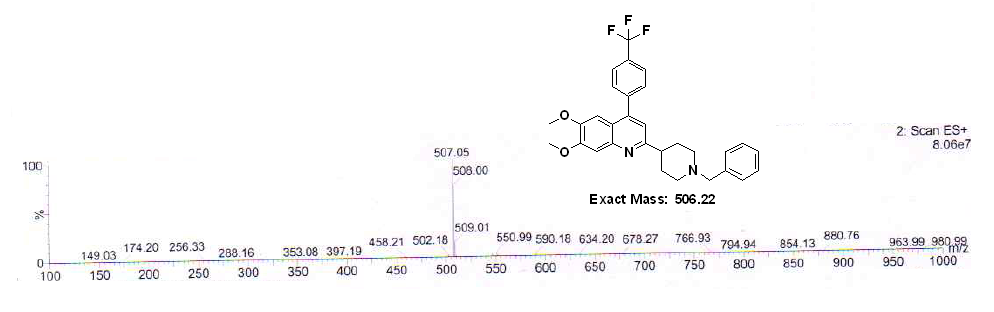
**

| **Calculated m/z** | 506.22 | **Ion formula** | C_30_H_29_F_3_N_2_O_2_ | **Species** | [M+H]^+^ |
| --- | --- | --- | --- | --- | --- |
| **Result type (ESI mode)** | | | Target m/z | | |
| Positive ions | | | 507.05 | | |

**^1^H-NMR spectrum of compound-23 in DMSO-d_6_**

**
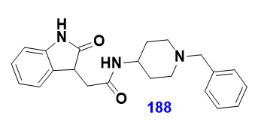

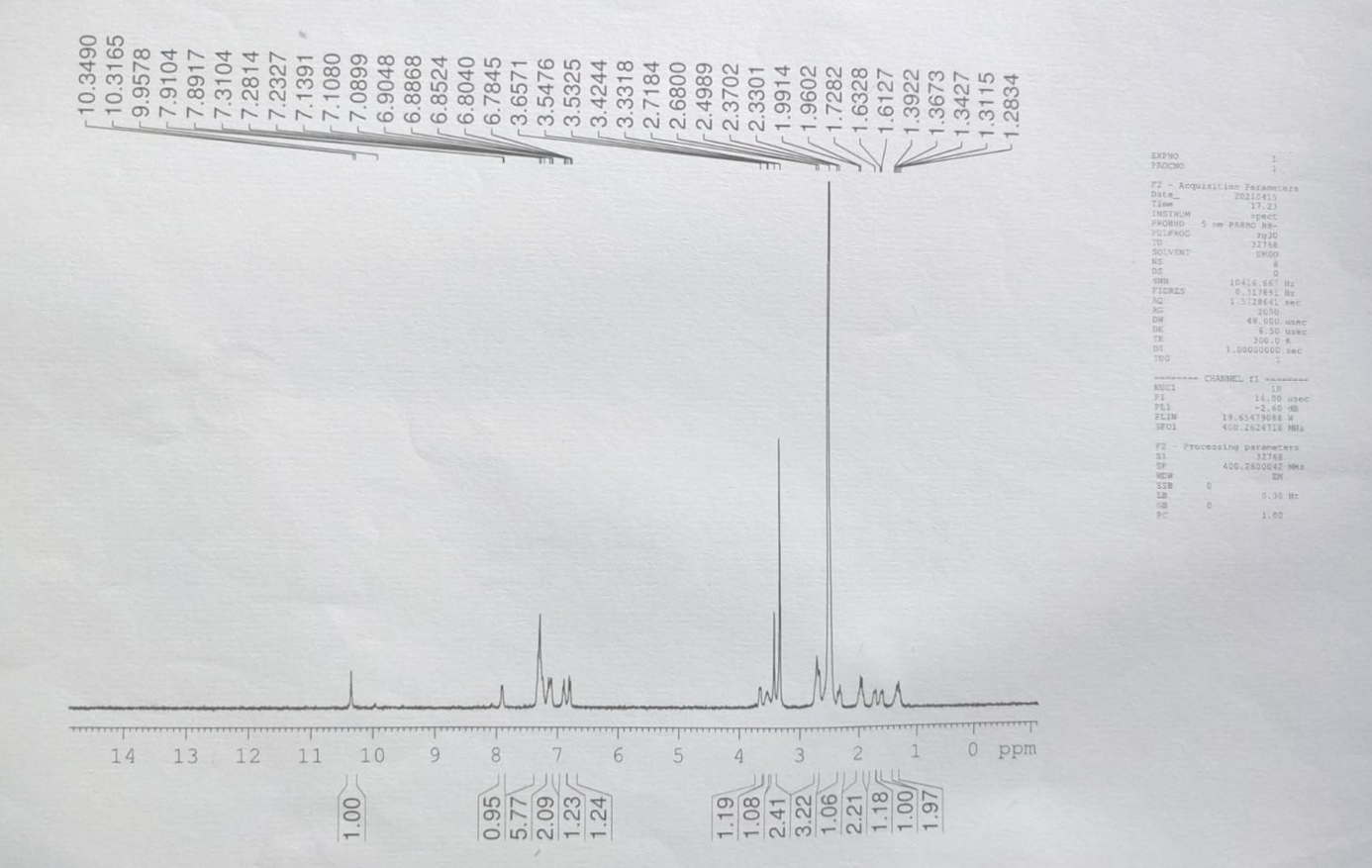
**

**Mass spectrum of Compound-23**

**
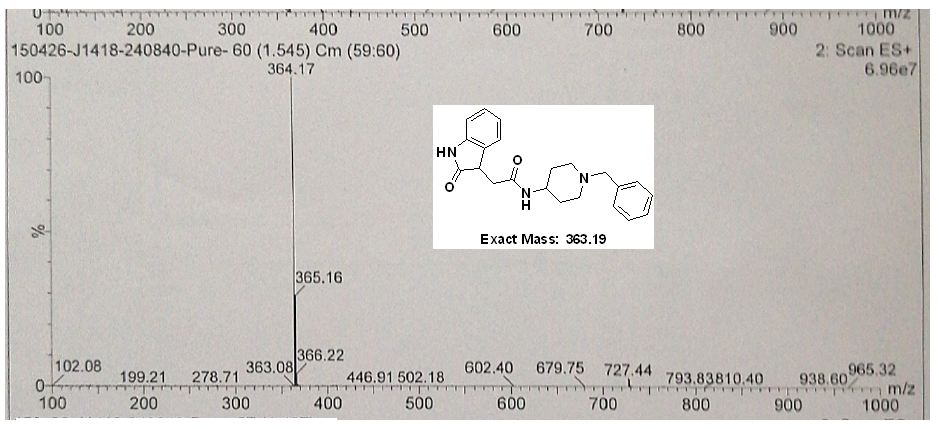
**

| **Calculated m/z** | 363.19 | **Ion formula** | C_22_H_25_N_3_O_2_ | **Species** | [M+H]^+^ |
| --- | --- | --- | --- | --- | --- |
| **Result type (ESI mode)** | | | Target m/z | | |
| Positive ions | | | 364.17 | | |
